# Tracing Back the History of Pepper (*Capsicum annuum*) in the Iberian Peninsula from a Phenomics Point of View

**DOI:** 10.3390/plants11223075

**Published:** 2022-11-13

**Authors:** Cristina Silvar, Filomena Rocha, Ana M. Barata

**Affiliations:** 1 Grupo de Investigación en Bioloxía Evolutiva, CICA—Centro Interdisciplinar de Química e Bioloxía, Universidade da Coruña, 15071 A Coruña, Spain; 2 Banco Portugues de Germoplasma Vegetal (BPGV), Instituto Nacional de Investigaçao Agraria e Veterinaria (INIAV), 4700-859 Braga, Portugal

**Keywords:** *Capsicum*, germplasm diversity, landraces, high-throughput phenotyping, Tomato Analyzer, Spain, Portugal

## Abstract

The Iberian Peninsula was the place where pepper (*Capsicum annuum*) entered Europe and dispersed to other continents but was also an important secondary center for its diversification. The current work evaluated the phenotypic diversity existing in this region and investigated how that evolved from *Capsicum* native areas (Mexico and Andean Region). For that purpose, the high-throughput phenotyping tool Tomato Analyzer was employed. Descriptors related to size and shape were the most distinctive among fruit types, reflecting a broad diversity for Iberian peppers. These traits likely reflected those suffering from more intensive human selections, driving the worldwide expansion of *C. annuum*. Iberian peppers maintained close proximity to the American accessions in terms of fruit phenomics. The highest similarities were observed for those coming from the southeastern edge of the Peninsula, while northwestern accessions displayed more significant differences. Common fruit traits (small, conical) suggested that Portuguese and Spanish landraces may have arisen from an ancient American population that entered the south of Spain and promptly migrated to the central and northern territories, giving rise to larger, elongated, and blocky pods. Such lineages would be the result of adaptations to local soil–climate factors prevailing in different biogeographic provinces.

## 1. Introduction

Peppers are one of the most important vegetables worldwide due to their versatility for cuisine, medicine, and industry [1]. This crop belongs to the genus *Capsicum*, which harbors five domesticated species (*C. annuum* L., *C. chinense* Jacq., *C. frutescens* L., *C. baccatum* L., and *C. pubescens* Ruiz et Pav.) and almost 40 wild species [2]. The genesis of *Capsicum* apparently occurred in a broad area spanning Peru, Ecuador, and Colombia along the Andes in western–northwestern South America [3]. The subsequent expansion of the genus took place following a clockwise direction around the subcontinent, from Colombia to central–eastern Brazil, Paraguay, northern Argentina, Bolivia, and Peru, returning to northwestern South America. Within this migration, Bolivia and Peru emerged as particularly important centers of diversification and origin of some cultivated species. Further spread of Andean peppers toward Central America gave rise to an Annuum clade including the *C. annuum* species, which was presumably domesticated in Mexico from the wild bird pepper *C. annuum* var. *glabriusculum* [3,4]. Following the voyages of Columbus in the late 15th century, peppers were introduced to Europe after their initial arrival in the Iberian Peninsula. Then, trade routes promoted their geographic dispersal toward the Mediterranean Basin, and thenceforth to Africa, India, and China [5]. *Capsicum annuum* was the most successful in this conquest, probably due to it being the first *Capsicum* species to arrive in Europe rather than related to any superior agronomic trait [6]. Currently, this species holds the greatest economical recognition, being grown in most tropical, Mediterranean, and temperate regions of the world [7]. Contemporary *C. annuum* has both sweet and hot types and encompasses some of the most widely consumed pepper varieties including bell pepper, jalapeno, cayenne, and poblano.

The Iberian Peninsula emerged as a highly relevant secondary diversification center for pepper as for other crops that originated in America (i.e., tomato) or the Fertile Crescent (i.e., barley) [8]. The variable orography, diverse climates, and edaphic versatility of this region promoted the generation of hundreds of landraces that were selected for over five centuries by farmers to fit distinctive environments and local consumption habits, resulting in an awesome phenotypic diversity of pepper native varieties [9]. Rivas-Martínez et al. [10] defined two regions (Eurosiberian and Mediterranean) and eight biogeographic provinces in the Iberian Peninsula: Pyrenean, European Atlantic (EA), Valencia-Provençe and Balearic (VPB), Central Iberian Mediterranean (CIM), West Iberian Mediterranean (WIM), Murcia–Almeria (MA), Betica (B), Coastal Lusitania, and West Andalusia (CLWA). The Eurosiberian region extends along the north of Spain and northwest of Portugal, and it coincides with the oceanic or Atlantic climate zones. Those areas are characterized by high and regular rainfall, mild temperatures, and humid summers. The Mediterranean region occupies 80% of the Peninsula and the Balearic Islands, covering the Mediterranean climate zones. It is distinguished by scarce and irregular rainfalls, warm temperatures, and hot and dry summers. The Canary Islands (CI) are part of the Macaronesian region where a subtropical climate prevails, with warm temperatures throughout the year and low rainfall [11]. A considerable number of ancient pepper landraces persist in cultivation in Spain and Portugal, contributing greatly to the subsistence and economic sustainability of regional smallholder communities [12]. They are consumed as fresh vegetables or employed to produce pepper powder (*Pimenton*, in Spanish) or canned food. Within such an extensive plant genetic reservoir, a great variability of shapes, sizes, and colors can be found, ranging from tiny hot peppers (*Pimiento de Padron*) to highly elongated pods (*Guindilla de Ibarra*, *Jaranda*), as well as round (*Bola*), triangular (*pimiento del Piquillo*), and sweet blocky/rectangular types (*Pimiento Morrón*) [13]. In fact, various pepper landraces from different regions provided the genetic basis for the development of well-defined ecotypes that currently possess Protected Designations of Origin (PDO) [14]. Iberian pepper landraces are also highly appreciated by consumers, who increasingly demand tastier vegetables with greater nutritional values [15].

Genetic variability constitutes the essential source of any breeding progress. Therefore, efforts to conserve and characterize the diversity of pepper germplasm collections are mandatory tasks to address future global demand for food, respond to climate change, and guarantee agrodiversity [16]. For this reason, the identification of the traits that suffered from the strongest modifications during hundreds of years of diversification in primary and secondary centers becomes relevant. Fruit morphometric parameters such as size, shape, weight, and color represent the attributes likely subjected to higher selection pressures imposed by humans [17]. Indeed, they are currently critical for varietal recognition, the establishment of suitable market niches, or the acceptance of new pepper varieties by consumers [18]. Therefore, an extensive and detailed assessment of fruit phenomics is essential to discern the diversity available for breeding and the evolution of pepper types from their native areas. Morphological evaluations of pepper fruits were traditionally based on highly heritable standardized descriptors related to specific characters or agronomic qualities [19]. However, such conventional characterization often entails multiples obstacles associated with time-consuming, lower dissection of trait architecture. or strongly biased estimations [20]. High-throughput automated phenotyping overcomes these limitations, revealing more effective systems to perform accurate phenomics assessments. Among these tools, the Tomato Analyzer (TA) software is a freeware that allows fast, precise, and semi-automatic dissection of a large set of quantitative traits from scanned images of fruit sections [21,22].

Despite its relevance as a secondary center for *C. annuum* diversification, pepper genetic resources from the Iberian Peninsula are mostly unexplored. Only a few studies have addressed the genetic characterization of a limited number of Spanish landraces [12,23,24]. In addition, a reduced number of reports employed those local accessions to assess the fruit flavor, nutritional composition, or quality of pepper powder [25,26]. Recently, Pereira-Dias et al. [27] used conventional descriptors and digital parameters to characterize a collection of pepper heirlooms and landraces from Spain. However, that study did not consider any germplasms from Portugal, which are practically unknown at the genetic and phenotypic levels. It is probable that native varieties from the Iberian Peninsula preserve a higher portion of the genetic wealth stored in primitive plants from America and have even enlarged that genetic asset after adaptation to multiple environments in Spain and Portugal. This makes Iberian pepper accessions a promising resource for the breeding of new market types with added value. The principal goal of this work was to assess the diversity of pepper fruit phenomics from the different biogeographic provinces in the Iberian Peninsula and investigate how that evolved from *Capsicum* native areas (Ecuador, Peru, and Bolivia) and domestication points such as Mexico. Pepper morphologies predominant in the Mediterranean Basin were also considered for comparative purposes, as this region might comprise an additional diversification spot in the migration of *C. annuum* to other continents.

## 2. Results

### 2.1. Digital Analysis

Seven traits (DEC, PEC, HAob, DIA, PIA, DEP, and Ob) that did not display a normal distribution were removed before the analysis of variance. Highly significant differences (*p* < 0.001) were observed among the means of accessions for the other 40 Tomato Analyzer descriptors and two conventional traits (Table 1). Considering the F-value, the parameters that most contributed to explain the variance were those within the basic measurement and fruit shape index categories. Among these, the greatest variability, according to the coefficient of variation, was found for FWE (117.3%), with fruits ranging from 0.10 (*C. annuum* var. *glabriusculum* PM647) to 404.3 g (Portuguese accession BPGV12438). The analysis of color space also revealed variations among accessions with larger amounts of redness and yellowness. Thus, average Hue ranged from 44.4 to 101.9, suggesting that fruit colors varied from red-orange to yellow-green. Similarly, mean ALV (46.4%) and ACh (48.0%) values indicated that the majority of peppers displayed moderate lightness and saturation of colors.

**Figure 1:**
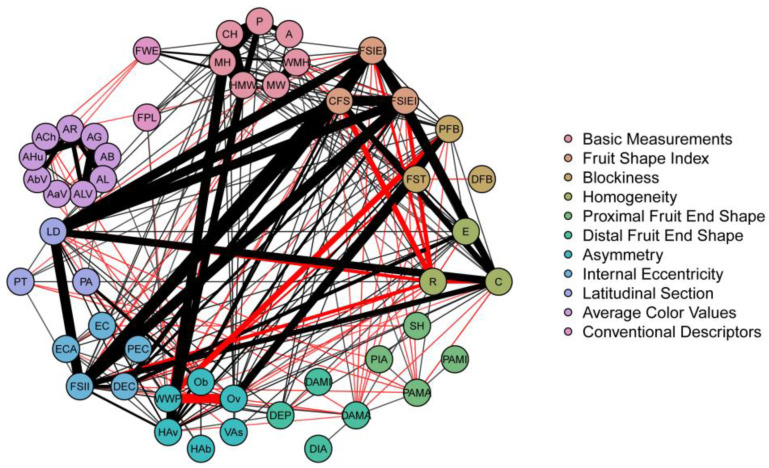
Correlation network depicting relationships among fruit descriptors. Acronyms can be found in Table S5. Each specific color represents one of the 10 categories described by TA software and the two analyzed conventional parameters (FWE and FPL). The width of bands is proportional to the correlation coefficients. Positive correlations are shown in black, while negative ones appear in red. Only significant coefficients (*p* < 0.05) higher than ±0.5 are represented.

Interdependences between TA descriptors were investigated using a correlation matrix (Figure S1). The significant interactions after Bonferroni correction (*p* < 0.05) between closely related (r > ±0.5) parameters were depicted by a correlation network (Figure 1). Fresh weight (FWE) displayed strong and positive correlations with parameters defining fruit magnitude (A, WMH, MW, and R), but negatively correlated with fruit shape indices (FSIEI, FSIEII, and CFSI), although the latter were not significant. Fruit size traits tightly interacted among themselves, but also with various shape descriptors, primarily FSIEI, FSIEII, CFSI, Ov, HAov, FSII, and LD. The categories fruit shape index, blockiness, homogeneity, internal eccentricity, and asymmetry demonstrated strong and positive correlations for every trait except rectangular. Colorimetric parameters were distinctly separated from the other TA groups and primarily interconnected among themselves, except for a few negative correlations established with fruit fresh weight (Figure 1).

### 2.2. From America to the Iberian Peninsula and the Mediterranean Basin

Variations in fruit morphology between American (Mexico and Andean Region) and Afro-Eurasian (Iberian Peninsula and Mediterranean Basin) countries were investigated using an analysis of variance. Highly significant differences (*p* < 0.001) were observed for 34 out of 42 traits, while no significant differences were detected for PAMI, AG, and AHue (Table S1). The attributes that primarily explained the variance were those related to fruit size and shape, i.e., FWE and those in the categories of basic measurements (P, A, WMH, and MW), shape index (FSIEI), homogeneity (C), and latitudinal section (PA).

**Figure 2:**
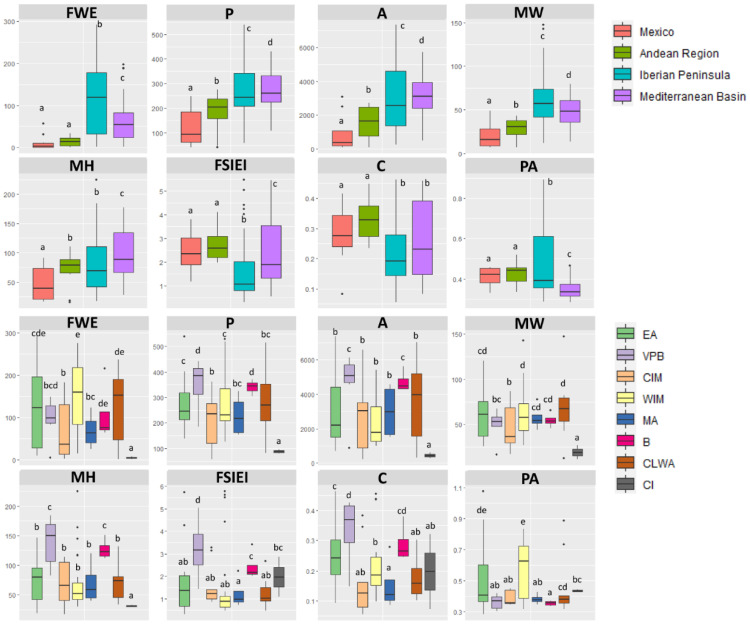
Box plots for the most distinctive parameters among the global and Iberian geographic regions. We show the 25th percentile, median (thick line), 75th percentile, and range minimum–maximum. Outliers (black circles) were identified as 1.5 times the interquartile range. Different letters indicate significant differences at *p* < 0.05. EA = European Atlantic, BP = Betica Province, CIM = Central Iberian Mediterranean, CLWA = Coastal Lusitania and West Andalusia, MA = Murcia and Almeria, VPB = Valencia-Provence and Balearic, WIM = West Iberian Mediterranean, CI = Canary Islands.

A detailed inspection of the most variable descriptors revealed that Mexico and the Andean Region possessed lighter fruits with lower variability, while the heaviest appeared in the Iberian Peninsula, which also exhibited the greatest fluctuation for the fresh weight parameter (Figure 2). The largest fruits, when considering perimeter, area, and MH, were observed in the Mediterranean Basin, although the widest were detected in the Iberian Peninsula. According to the FSIEI and circular traits, peppers from Mexico and the Andean Region displayed less rounded outlines than those in the Iberian Peninsula and Mediterranean Basin. Pericarp area, which indicates fruit wall thickness, suggested that Iberian peppers were fleshier than those from other geographical areas.

Divergences in fruit characteristics among the eight biogeographic provinces described in the Iberian Peninsula were also explored. In this case, one trait (R) was recorded as not significant, whereas 31 were recorded as highly significant (*p* < 0.001) (Table S2). As shown previously, parameters describing fruit size and shape represented those with higher distinctiveness among regions. Thus, peppers from the Canary Islands (CI) showed the smallest weights (FWE), while those from the West Iberian Mediterranean Province (WIM) exhibited the weightiest fruits, although the latter was not significantly different from the CLWA, B, and EA provinces (Figure 2). The Canary Islands also comprised the fruits with the lowest perimeter (P), area (A), maximum width (MW), and maximum height (MH), whereas the peppers with the largest surfaces were found in the Betica (B), Valencia-Provençe, and Balearic (VPB) provinces. In fact, the latter also possessed the fruits with highest FSIEI and lowest circular values, indicating that elongated peppers prevailed in these Spanish geographical areas. On the contrary, more rounded fruits of medium size and weight were most common in the Murcia-Almeria (MA) province. The thickest pericarps (PA trait) were detected in the WIM and EA regions, while the thinnest were detected in the Betica, VPB, MA, and Central Iberian Mediterranean (CIM) provinces (Figure 2).

**Figure 3:**
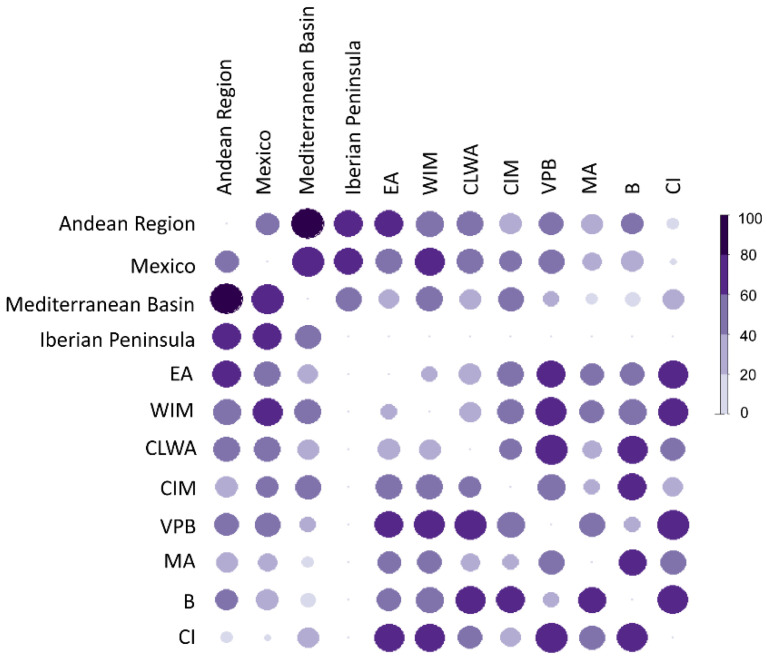
Percentage of pairwise significantly different traits (*p* < 0.05) among global and Iberian geographic regions. The color intensity and the size of circles are proportional to the percentage of divergences. EA = European Atlantic, BP = Betica Province, CIM = Central Iberian Mediterranean, CLWA = Coastal Lusitania and West Andalusia, MA = Murcia and Almeria, VPB = Valencia-Provence and Balearic, WIM = West Iberian Mediterranean, CI = Canary Islands.

The percentage of pairwise significantly different traits was calculated to illustrate how geographical regions differed from each other according to pepper fruit morphometrics (Figure 3). Mexican and Andean peppers exhibited the lowest number of pairwise differences (41.9%), indicating that fruits from these regions had a comparable morphology. The Iberian Peninsula displayed a total of 27 out of 41 (65.8%) significant pairwise differences with both Mexico and the Andean Region, while the greatest similarity (51.2% of differences) appeared with the Mediterranean Basin. The analysis of divergences among the eight biogeographical regions in the Iberian Peninsula revealed that fruits from the Canary Islands possessed great similarity with those from America (Mexico and Andean), as they displayed the lowest percentage of different traits (4.5% and 11.4%, respectively). On the contrary, peppers from the West Iberian Mediterranean (WIM) and European-Atlantic (EA) provinces showed the highest number of pairwise differences with Mexican (65.9%) and Andean (61.4%) fruits, respectively (Figure 3). In turn, countries in the Mediterranean Basin encompassed pepper fruits that highly resemble those from the Murcia-Almeria (MA) (11.4% of different traits), Betica (B) (18.2%), and Valencia-Provençe and Balearic (VPB) (20.5%) areas. If considering only the Iberian Provinces, the most comparable (20% of pairwise dissimilarities) pepper fruits were found between WIM and EA, between B and PB, and between M and CIM. On the other hand, the regions with the highest mean percentage of significantly different fruit traits were Balearic (VPB) (61%) and Canary Islands (CI) (59%) (Figure 3).

### 2.3. Evolution of Pungency

**Figure 4:**
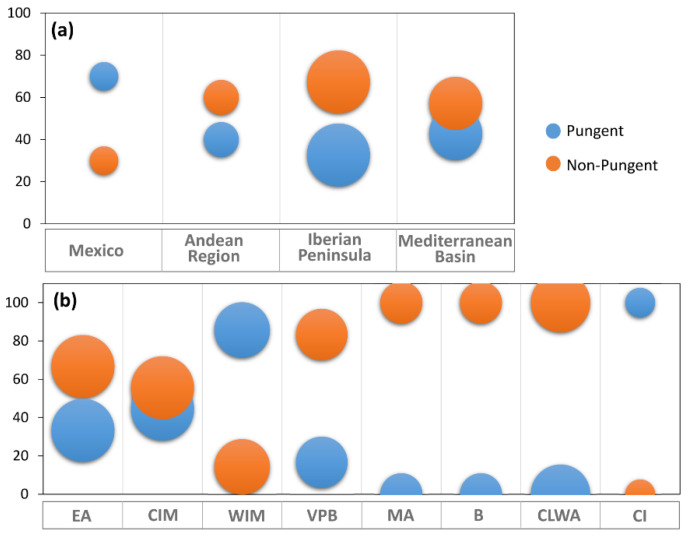
Percentage of pungent and non-pungent accessions from the different global (**a**) or Iberian (**b**) geographical regions. The size of the circles is proportional to the number of accessions within each geographical group. EA = European Atlantic, BP = Betica Province, CIM = Central Iberian Mediterranean, CLWA = Coastal Lusitania and West Andalusia, MA = Murcia and Almeria, VPB = Valencia-Provence and Balearic, WIM = West Iberian Mediterranean, CI = Canary Islands.

Variations in the pungency trait were assessed to establish differences between the primary and secondary diversification centers. Mexico was the region with the highest percentage of plants with pungent fruits (70%), while the Iberian Peninsula mostly comprised non-pungent accessions (67.4%) (Figure 4a). The ratio between sweet and hot peppers was lower in the Andean region and Mediterranean Basin, although the non-pungent types prevailed over the others. The analysis of pungency in the different biogeographical regions of the Iberian Peninsula brought out the total absence (0%) of pungent fruits in the Betica (B), Coastal Lusitania and West Andalusia (CLWA), and Murcia-Almeria (MA) areas. On the contrary, hot peppers represent the majority in the Canary Islands (100%) and the West Iberian Mediterranean (WIM) province (85.7%) (Figure 4b).

The analysis of variance showed that 10 out of 42 fruit descriptors were not significantly different between pungent and non-pungent types, while 27 traits exhibited significant differences at *p* < 0.001 (Table S3). Broader variability, expressed as coefficients of variation, was found within the pungent group, in which CVs higher than 50% were recorded for up to 17 traits. On average, sweet peppers had weightier, huger fruits with more rectangular or rounded shapes, thicker walls, and less redness and bright colors (Table S3).

### 2.4. Multivariate Analysis

Eight traits (HMW, WMH, CH, FSIEI, CFSI, LD, FSII, and HAov) displaying coefficients of correlation higher than 0.9 with at least two other parameters were not considered for the principal component analysis. PCA showed that eight components displayed eigenvalues > 1, cumulatively accounting for 83.43% of the variance. The first component (PC1) explained 27.18%, and it was positively and robustly correlated (>0.8) to TA attributes related to shape such as FSIEI and Ov. The second component (PC2) accounted for 17.82% of the variance, which was mostly contributed by three size traits (P, A, and MW) with positive correlations above 0.8 (Figure S2).

**Figure 5:**
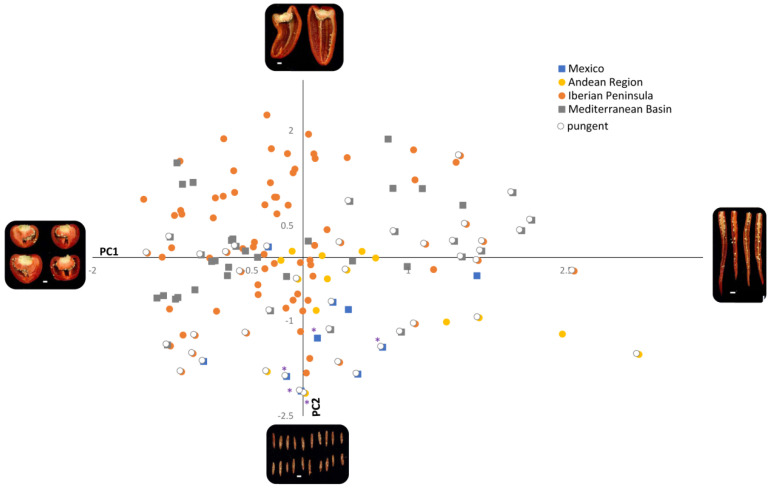
Scatter plot for the PCA analysis performed on the whole collection. Pungent peppers are highlighted with a white circle. Purple asterisks indicate *C. annuum* var. *glabriusculum* accessions. Pictures represent the accessions at the outermost edges of each component. White lines inside the photos indicate a 1 cm scale.

The two principal components were used to project the pepper accessions on two-dimensional plots. They were tagged according to the geographical regions considered in this work. In general, pepper fruits were widely dispersed on the PCA diagrams according to their shape and size (Figure 5, Table S4). Hence, South American accessions were primarily plotted in the quadrant defined by positive PC1 and negative PC2 axes, denoting that those peppers possessed highly elongated shapes (FSIEII = 5.99, C = 0.43, FSII = 5.74, and LD = 51.89) and small sizes (*p* = 40.72, A = 85.90, and MH = 16.38). The smallest fruits corresponded to accessions belonging to wild *C. annuum*. Pepper fruits from the Iberian Peninsula mostly concentrated on the negative panel of PC1. In this way, fruits from this region varied from highly elongated morphologies with large height/width ratios (FSIEII = 10.89, C = 0.46) to strongly roundish fruits (FSIEII = 0.84, C = 0.09). Similarly, size varied from extremely huge fruits with areas of 7334.01 mm^2^ to much smaller berries (A = 229.17 mm^2^). Despite this, most Spanish and Portuguese peppers included in this work displayed large/medium dimensions (average A = 3006.07 mm^2^) and triangular/rectangular contours (average FSIEII = 2.14, C = 0.23). Mediterranean peppers exhibited the widest dispersion, with accessions showing positive and negative values for both the first and the second components. Very elongated peppers were found in Bulgaria (CAP491, FSIEII = 10.44, C = 0.46) and Turkey (CGN24358, FSIEII = 8.89, C = 0.45), while the most rounded types (FSIEII = 1.27, C = 0.12) appeared in Eastern European countries (Hungary, Bulgaria, and former Yugoslavia). Likewise, the largest fruits (A = 5723.18 mm^2^, MH = 170.29) were recorded in the European Mediterranean region (France, Italy, and Albania) in contrast with the tiniest ones (A = 540.18 mm^2^, MH = 42.43) observed in African Mediterranean countries (Tunisia and Libya). The pungency trait was also influenced by the distribution of pepper accessions on the graphical space. Thus, hot types appeared in the areas bounded by the positive PC1 and negative PC2 axes, denoting that pungent fruits possessed an elongated shaped and medium to small sizes (Figure 5).

**Figure 6:**
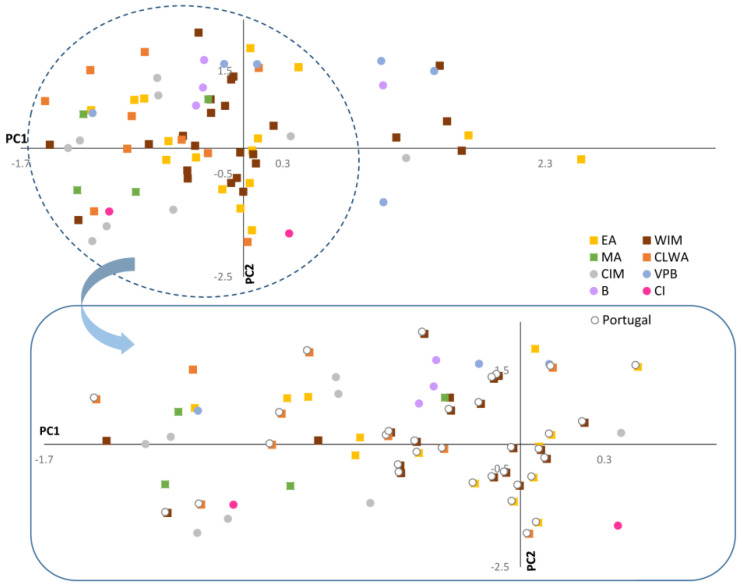
Scatter plot for the PCA analysis performed on the Iberian collection. Portuguese peppers are highlighted with a white circle. EA = European Atlantic, BP = Betica Province, CIM = Central Iberian Mediterranean, CLWA = Coastal Lusitania and West Andalusia, MA = Murcia and Almeria, VPB = Valencia-Provence and Balearic, WIM = West Iberian Mediterranean, CI = Canary Islands.

An exhaustive analysis of the arrangement of Spanish and Portuguese accessions on the graphical space was performed to investigate associations between biogeographic provinces and fruit morphometrics (Figure 6). However, the allocation of Iberian peppers did not follow a geographical pattern, and accessions from the Iberian Peninsula primarily converged on the negative segment of PC1, which indicated that the majority of peppers from this region exhibited blocky, rectangular, conical, or round shapes. Nevertheless, various elongated peppers were also identified, mainly in the WIM, EA, and VPB provinces. Interestingly, these long fruits were recorded in Spain, but not in Portugal. Iberian accessions showed wide variation in fruit size, as demonstrated by their dispersion throughout the area defined by the second component (Figure 6). Thus, the fruit descriptor area oscillated between 7334.01 mm^2^ (OI2705) and 229.17 mm^2^ (BGHZ0280). Large, medium, and small peppers were distributed throughout all biogeographic regions except for B and VPB, where huge fruits were predominant.

**Figure 7:**
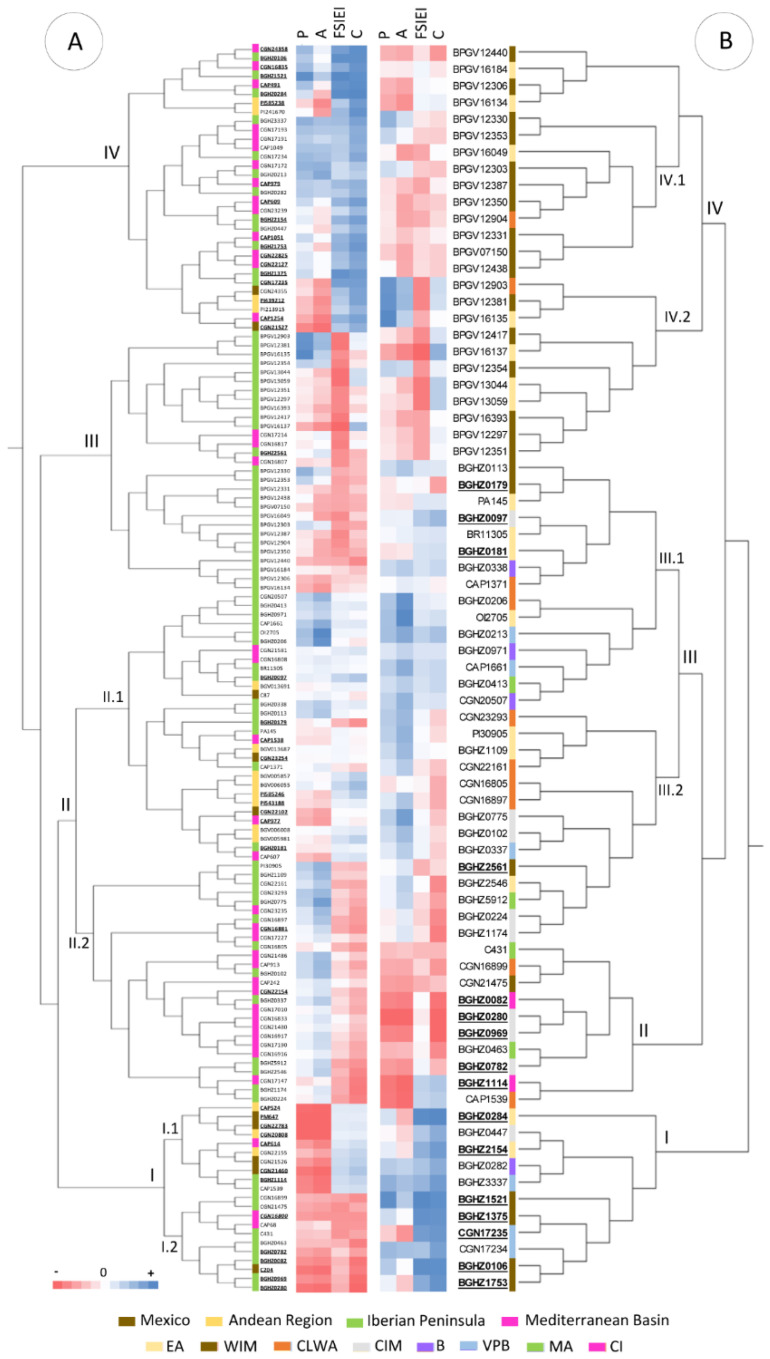
Cluster analysis for global (**A**) and Iberian (**B**) accessions. The heatmap represents the variables standardized to z-scores. EA = European Atlantic, BP = Betica Province, CIM = Central Iberian Mediterranean, CLWA = Coastal Lusitania and West Andalusia, MA = Murcia and Almeria, VPB = Valencia-Provence and Balearic, WIM = West Iberian Mediterranean, and CI = Canary Islands. Pungent accessions are in bold and underlined.

Groups of accessions with common traits were identified with a hierarchical cluster analysis by making use of those parameters with large contributions to the ANOVA and PCA variances (Figure 7). The tiniest fruits were positioned in cluster I, which was primarily comprised of peppers from the Iberian Peninsula (47.6%) and America (38.1%). Two well-defined subgroups, according to fruit shape parameters (FSIEI, C), could be established within this cluster. Subgroup I.1, mostly represented by Iberian accessions (72.7%), displayed circular forms, while subgroup I.2, containing 70% of American peppers, exhibited conical shapes and included the Mexican wild species *C. annuum* var. *glabriusculum* PM647, CGN21526, CGN22783, and CGN20808 (Figure 7A). Cluster II hosted the majority of the total analyzed accessions (41%) and could be divided into two subclusters, both of them displaying fruits of medium–large magnitude. Subgroup II.1 comprised triangular types from the Iberian Peninsula (46.7%), America (36.7%), and the Mediterranean Basin (16.6%), while subgroup II.2 harbored rectangular and blocky forms, all of which came from the Iberian Peninsula (48.2%) and Mediterranean countries (51.8%). The third cluster (III) harbored the fruits with the most rounded shapes and medium sizes, primarily represented by Portuguese accessions. Lastly, the most elongated fruits were detected in cluster IV, which was composed of accessions from the Mediterranean region (43.8%) and the Iberian Peninsula (37.5%). This cluster also encompassed the fruits with the largest perimeters and areas, except for a reduced subgroup, which comprised American peppers of smaller sizes (CGN24355, PI439212, PI213915, and CGN21527). *Capsicum annuum* with hot fruits were mostly circumscribed to cluster IV (43.2% of total pungent fruits), but also appeared in clusters I (29.5%) and II.1 (18.2%) (Figure 7A). The analysis of the grouping in the Iberian set arranged accessions into four clusters according to fruit morphometrics (Figure 7B). Cluster I, which was the most distant, consisted of highly elongated peppers from the WIM (36.4%), VPB (27.3%), and EA (18.2%) regions. The second cluster (II) comprised 10 accessions broadly dispersed across CIM (30%), CLWA (20%), MA (20%), and the Canary Islands (CI, 20%). Peppers in this group displayed the smallest perimeters and areas while showing conical and rounded forms. Accessions in cluster III exhibited a wide distribution over all biogeographic regions in Spain, although a substantial proportion (48%) converged at the most eastern edge of the Iberian Peninsula (Valencia, Murcia, Balearic, and Andalusian areas) or at the Cantabrian and Atlantic areas of Spain and Portugal (34.5%). This group harbored the largest fruit sizes with two distinguishable morphologies that allowed its partition into subgroup III.1 and III.2. The former mostly consisted of peppers with triangular shapes, whereas the latter hosted blocky and rounded fruits. Lastly, Portuguese accessions were primarily grouped in cluster IV, and they contained medium-size fruits displaying either square–roundish shapes (sub-group IV.1) or triangular–oblong forms (IV.2). Accessions in those groups extended over the West Iberian (WIM, 64%) and European Atlantic (EA, 28%) provinces. Clusters I and II concentrated the highest number of pungent types, with 43.8% and 31.3%, respectively (Figure 7B).

## 3. Discussion

Thousands of pepper landraces are currently stored in germplasm banks around the world. Beyond the conservation of these resources, their exploitation depends on our ability to characterize them. Apart from an adequate genetic assessment of diversity, a detailed evaluation of plant and fruit phenomics will be essential. Fruit weight, size, shape, and color largely influence the potential of a particular landrace to be introduced into breeding programs or directly recovered for new target markets [18]. On the basis of phenotypic conventional descriptors, the plant breeding process has usually focused on large screening tests of a specific trait to trap the alleles of interest. However, newly developed digital tools for high-throughput phenotyping made possible the extremely accurate estimation of many fruit attributes simultaneously, many of which are nearly impossible to obtain manually or visually [20]. Such approaches will accelerate precision breeding for distinctive fruit traits, but also the easy tracking of allelic variants associated with phenotypic variations directly within collections of nonredundant genetic materials as performed by association analyses [28].

Worldwide pepper landraces likely maintain significant combinations of traits that were left behind when growers aimed for modern cultivars. It is predictable that native varieties from the centers of origin and diversification, such as Mexico, the northwestern Andes (Ecuador, Peru, and Bolivia), the Iberian Peninsula, and the Mediterranean Basin, preserved a higher portion of the wealth stored in primitive *Capsicum* spp., constituting a promising resource for the development of new elite genitors. Despite such relevance, pepper germplasms from those areas are poorly investigated at the phenomics level. Only a reduced number of works have focused on the morphological characterization of landraces from Mexico, Ecuador, or the Andean region using both conventional and digital descriptors [29,30,31,32,33,34]. Recently, Pereira-Días et al. [27] employed conventional and high-performance imaging tools (Tomato Analyzer) to morphologically characterize a collection of heirlooms, mostly originating in Spain and preserved at the Institute for Conservation and Improvement of Valencian Agrodiversity (COMAV). To the best of our knowledge, no report thus far has attempted to explore the phenotypic variability of traditional pepper varieties from Portugal. In the present work, a set of 139 *C. annuum* accessions comprising mainly old Spanish and Portuguese landraces was evaluated for fruit phenomics. Many of them are still cultivated today, exemplifying a valuable source of unexploited variability in a cultivated background. Attention was also paid to the diverse biogeographic provinces existing in the Iberian Peninsula, which considerably differ in climate, soil, and altitude, factors that might affect the distribution of cultivated peppers.

The analysis of variance found that descriptors related to size, shape, and fresh weight were those that contributed most to explaining variability within fruit types. Comparable results were observed in our and other previous works on pepper [17,33,34,35], as well as on tomato [36,37] and eggplant [38]. Similarly, basic measurements and fruit shape indices exhibited the highest contributions to the principal component approach, explaining up to 50.84% of the fruit variability. In this case, shape, homogeneity, and the internal eccentricity descriptors FSIEI, FSIEII, CFSI, C, DEC, FSII, and LD were more powerful than the size attributes P, A, HMW, MH, and CH for determining the distribution of accessions on the biplot. The remarkable influence of shape, size, and color for capturing pepper diversity was already brought to light by Pereira-Dias et al. [27] and Nankar et al. [39] in collections of Spanish and Balkan accessions, respectively. Basic measurements and fruit shape indices are essential parameters for the commercialization of new pepper varieties and culinary usages [40]. Fruit height and width will determine the classification of pepper fruits into different commercial categories (small, medium, or large), whereas shape will considerably affect, for example, the packaging method [41].

Correlations between fruit descriptors revealed that fruit weight (FWE) was positively correlated with TA parameters related to size, especially width (P, A, WMH, and MW), but negatively correlated with fruit shape indices, internal eccentricity, and color attributes. Such results showed that heavier fruits possessed higher perimeters and areas but lower ratios for height to width, i.e., blocky shapes and duller red colors. Vilarinho et al. [42] also reported significantly positive correlations between fruit weight and width, supporting the data by Barchi et al. [43], who found that QTLs controlling those traits were linked on chromosome P12. As reported earlier [17,34,35], correlations observed among MW, FSIEI, EC, FSII, and C suggested that wider fruits displayed lower fruit indices but more roundish shapes. Significant associations among the categories of homogeneity, fruit shape index, and internal eccentricity were observed as well, corroborating data by Tripodi and Greco [35] on a large collection of cultivated and wild peppers. Connections between pepper fruit size and shape were recently attributed to various QTLs clustered in some hotspot regions of the pepper genome [44,45]. In agreement with Nankar et al. [37], significant interactions for color parameters estimated with both RGB and CIELab spaces were mainly observed among them, but not with the remaining TA categories, except for FWE, Ob, and PA. Our results confirmed that a reduced subset of TA descriptors within the categories of basic measurements, fruit shape index, homogeneity, or latitudinal sections is the most informative. Indeed, these indicators might be used alone to disclose the levels of diversity and differentiate among pepper pods regardless of the germplasm collection considered [17,27,33,34,35,39]. Such characters likely reflect the ones suffering from more intensive human selections after domestication and subsequent differentiations, driving *C. annuum* spread around the world [46]. These traits might be further exploited by breeders to overcome the numerous limitations associated with morphological conventional descriptors, and they could be valuable to establish easy-to-handle, accurate, and objective fruit descriptors standardized for germplasm identification, varietal typification, or even protection of new landrace-derived cultivars [47,48]. No less important, such precise digital traits will result paradigmatic for the successful application of genome-wide association studies, which often become constrained by the poor quality of phenotypic data [49].

Apart from a deep assessment of pepper genetic resources for morphological diversity, it becomes necessary to investigate the evolution of pepper fruit morphometries after migration from the centers of origin to the secondary diversification areas. From the wild Mexican ancestor *C. annuum* var. *glabriusculum*, fruit attributes have likely suffered from strong distinctive human selection pressures in different regions depending on the prevailing agro-climatic conditions and human practices [50]. In the current work, TA descriptors were also effective for discerning the evolutionary history of pepper phenomics. Accordingly, Mexico and the Andean region exhibited the largest number of fruit characters in common, and they comprised pungent, light, tiny berries with conical outlines and thin pericarps. On the contrary, peppers from the Iberian Peninsula shared most traits with those from the Mediterranean Basin, although they maintained close proximity to the American types. This is somehow foreseen when considering that Spain was likely the place of entrance for this crop into Europe and a succeeding contributor to its expansion across Mediterranean countries through the trade routes that connected Asia with the Old World [5,46]. Comparable results were obtained in our previous work [34], which concluded that certain divergences in fruit morphology among peppers from Mexico, Ecuador, and Peru responded to a geographic pattern. Such differences might be the perceptible outcome of genome variations that occurred between primary and secondary diversification areas [51]. Thus, geographical proximity should favor a higher gene flow and greater genetic similarities, which might give rise to common fruit traits, as recently shown by Pereira-Dias et al. [24] in Spanish and European *C. annuum* landraces. The influence of ecogeographic distributions on the patterns of genetic differentiation were also reported for other *Capsicum* spp. [52,53]. Pungency, the most important quality trait in peppers, also differed among geographic regions. Hence, the majority of pungent fruits were found in Mexico, and they mostly corresponded to the *C. annuum* var. *glabriusculum* accessions. Wild peppers were characterized by small, soft berries with high levels of pungency, while cultivated forms were selected toward large-fruited, fleshy, non-pungent types with blocky shapes (bell pepper), particularly in the Western world [54,55]. The relatively high percentage of hot accessions found in the Mediterranean areas was ascribed to African (Tunisia and Libya), Eurasian (Israel and Turkey), and Eastern European countries (Bulgaria and Hungary), corroborating the culinary preferences of those territories for pungent varieties [7]. Such morphological divergences between pungent and non-pungent types are in agreement with data from Hill et al. [44], who found 17 conserved regions across the genome of non-pungent peppers and reported that many of those overlapped with QTLs for fruit size and shape, but also with genes involved in the capsaicin biosynthesis.

Despite the relevant role played by the Iberian territories in the dispersion of the *Capsicum* crop, the origin and relationships between the Spanish and Portuguese landraces from different areas remained poorly investigated. The current work attempted to understand how farmers adapted pepper plants to local growing environments and how this conditioned the gradual change of fruit morphologies from the original Mexican wild pepper. Among the biogeographic regions described in the Iberian Peninsula, fruits from the Canary Islands displayed the highest similarity with American peppers. Nevertheless, such results must be considered with caution since this province is represented by a reduced number of accessions. Peppers from the most southeastern part of Spain (Murcia-Almeria and Betica provinces) also possessed high similarities with those from Mexico and the Andean Region, while the most distant derived from the north, west, and northwestern territories, comprising the EA and WIM provinces. It was not possible to establish explicit correspondences between phenotypic and biogeographic data, although some trends could be noticed. Thus, elongated, pungent, and thin peppers constituted a unique and well-defined group in both PCA and cluster analyses. They appeared at specific regions in the West Iberian, European Atlantic, and Valencia-Balearic provinces, and they matched with recognized landraces employed for pickling in Basque Country (*Guindilla de Ibarra*) or destined for powder manufacturing in Extremadura (*Ocal*) and Valencia (*Pebre*). The accessions with the smallest and roundest fruits were located at the southeastern edge of Spain, and they also had correspondences with well-known local peppers, such as *Ñoras*. Pungency was mostly associated with elongated and conical fruits, while it was nearly absent in the other Iberian peppers. In general, the largest fruits with blocky and rectangular forms appeared in Valencia, Murcia, Andalucia, and, to a lesser extent, in EA and WIM. These data agreed with our previous genetic analysis on Spanish peppers, which found distinctive genomic compositions that seemed to correspond with specific fruit sizes, shapes, and potential pungencies at particular locations [23]. Considering the similarities described above to the primary centers of origin, it could be concluded that ancient populations from America might have entered the south of Spain in post-Columbian times and promptly migrated to central and northern areas, where they suffered from local selection under particular soil–climate conditions. The absence of genetic information for Portuguese accessions did not allow speculation on the origin of those landraces. Recently, Tripodi et al. [46] proposed that the old Portuguese trade routes shipped peppers from Brazil to Africa, India, and China but they likely did not introduce pungent peppers to Europe, as these might represent a competitor for the valued black pepper (*Piper nigrum*) that the Portuguese empire imported from the Far East. In addition, peppers diffused by the Portuguese from their Brazilian colonies likely corresponded to small and hot *C. chinense* more than *C. annuum* [5]. Our clustering analysis based on fruit phenomics revealed the existence of a well-differentiated group of Portuguese peppers, dispersed mainly in the north/northwest areas and displaying large–medium sizes with roundish, triangular, or blocky morphologies. On the other hand, Portuguese accessions from the south exhibited smaller/conical forms and they appeared closely related to comparable southern Spanish fruits. These results suggest a plausible common origin from Iberian peppers at the south of the peninsula, giving rise to particular lineages in their subsequent migration to the central and northern territories. Indeed, it cannot be discarded that Portuguese landraces may have derived from Spanish ones, although further work would be needed to confirm such a hypothesis.

The current work confirmed the overall progression proposed to explain the domestication and consequent selections carried out in *C. annuum* in the different areas of diversification around the world [56,57]. Hence, the wild progenitor *C. annuum* var. *glabriusculum* possessed erect, small, deciduous, red-colored fruits with notable pungency. Continued modifications led to variable degrees of pungency, flavor, color, shape, fruit wall thickness, and drying ability, giving rise to cultivated peppers containing either small hot fruits or large-fruited sweet types. In general, the tendency was toward the development of large, non-deciduous, and pendant fruits, with great shape variation, tremendous increases in fruit mass, and absence of pungency, which currently represent the most economically important types. Recently, Tripodi et al. [46] concluded that such transitions from wild to cultivated types were likely linked to the history of human trading worldwide, driven by diverse cultures, consumer preferences, and country economies. The Iberian Peninsula represents a valuable example of the great diversity still persisting today and its utility to dissect the primary selection forces that guided the establishment of current pepper varieties. Ongoing works based on new generation genetic approaches and genome-wide association studies will enable us to further investigate Spanish and Portuguese peppers for the genomic regions underlying the most significant fruit-related traits responsible for actual phenomic variability.

## 4. Materials and Methods

### 4.1. Plant Material

A collection of 139 pepper accessions, encompassing 134 *C. annuum* and five *C. annuum* var. *glabriusculum*, were selected for this work. Seventy-five are landraces representative of the geographic diversity present in the Iberian Peninsula, and they are maintained at the Vegetable Germplasm Bank of Zaragoza (BGHZ, Zaragoza, Spain), the Agricultural Research Center of Mabegondo (CIAM, A Coruña, Spain), and the Portuguese Vegetal Germplasm Bank (BPGV, Braga, Portugal) (Table S5). Pepper accessions were selected to cover the three biogeographic regions and eight provinces described by Rivas-Martínez et al. [10] in the Iberian Peninsula (Figure S3). The other 64 accessions exemplified the diversity existing in the *Capsicum* domestication centers (Mexico and the Andean Region) and secondary diversification regions (Mediterranean Basin). The latter represented different areas from Europe (Albania, Bulgaria, Former Yugoslavia, France, Hungary, Italy, and Slovenia), Africa (Libya and Tunisia), and Asia (Israel and Turkey). Most accessions were landraces, although some breeding lines were also included. All accessions were kindly provided as seeds by the Center for Genetic Resources (CGN, The Netherlands), the USDA-ARS Plant Genetic Resources Conservation Unit (USA), the Leibniz Institute of Plant Genetics and Crop Plant Research (IPK, Germany), and the Institute for Conservation and Improvement of Valencian Agro-diversity (COMAV, Spain). In addition, accessions were classified as pungent or non-pungent according to the information deposited in the Genebanks (if available) or according to the results of marker MAP1, linked to *Pun1* gene, as previously described [58] (Table S5).

Portuguese peppers were cultivated in a greenhouse during 2020 at the Instituto Nacional de Investigaçao Agraria e Veterinaria (S. Pedro de Merelim, Braga, Portugal) (41°34′ N, 8°27′ W). Trials were comprised of 10 plants per accession organized in randomized blocks. The other accessions were grown in a greenhouse in 2018 at the Centro de Investigaciones Agrarias de Mabegondo (Mabegondo, A Coruña, Spain) (43°15′ N, 8°18′ W). Four plants per accession were allocated following a completely randomized design. Plants were drip-irrigated and fertilized with a mix of nitrogen, phosphorus, and potassium before and after transplanting. Phytosanitary treatments against whiteflies, aphids, and spider mites were applied when necessary.

### 4.2. Fruit Phenomics Assessment

Ten to 25 mature fruits per accession (similar number for each plant) were harvested and subjected to digital phenotyping using the Tomato Analyzer v3 software [21,22]. Thus, fruits were longitudinally cut and scanned with an HP Scanjet G3110 photo scanner (Madrid, Spain) at a resolution of 300 dpi. Stored images (TIF format) were subsequently analyzed for 47 different morphometric and colorimetric descriptors categorized as basic measurements (7), fruit shape index (3), blockiness (3), homogeneity (3), proximal fruit end shape (4), distal fruit end shape (4), asymmetry (6), internal eccentricity (5), latitudinal section (3), and fruit color traits (9) (Table S6). A complete description of morphometric and colorimetric descriptors can be found elsewhere [21,22,59]. Default settings were used for all categories, although points were adjusted manually when the software was unable to accurately identify the outline of a trait. Two conventional traits, fruit weight (FWE) and fruit pedicel length (FPL), were also recorded according to IPGRI *Capsicum* descriptors [19].

### 4.3. Statistical Analyses

Data derived from processed images were statistically examined using SPSS v27 [60] and R v4.0.0 [61]. Fruit values (*n* from 10 to 25) for each digital descriptor (2193 total data points per trait) were considered as cases for statistical analyses. Means, standard deviations, and coefficients of variation (expressed in percentages as the ratio between standard deviation and mean) were used for descriptive analyses of traits. Boxplots were created with the *ggplot2* package [62]. Data were log-transformed, and the analysis of variance (ANOVA) was performed to detect differences among accessions and among the means of geographical regions. Kolmogórov–Smirnov and Levene tests were employed to check normality and homoscedasticity, respectively. Significant differences were discovered using post hoc Tukey HSD tests (*p* < 0.05). A correlation matrix between TA descriptors was estimated using Pearson’s test at *p* < 0.05 after Bonferroni’s adjustment for multiple comparisons [63]. The calculation of coefficients was performed with the *psych* [64] and *corrplot* [65] packages, while the correlation network was built using *qgraph* [66]. TA fruit descriptors were comprehensively examined through a multivariate approach using principal component analysis (PCA). For that purpose, highly correlated variables that displayed r > ±0.9 with at least two other parameters were removed beforehand. Likewise, outliers were previously detected and excluded. For the sake of clarity, scatter plots derived from PCA were depicted using the centroid (average) values for each accession. The similarity across accessions was estimated through the *factoextra* [67] package by employing an agglomerative hierarchical cluster analysis (HCA) with Euclidean distances and Ward’s coefficient. The graphical representation of the tree was performed with MEGA X software [68]. A heatmap was created using the package *gplots* [69] by standardizing the variables to z-scores.

## 5. Conclusions

Digital phenotyping based on Tomato Analyzer software was employed to characterize pepper fruit phenomics in a collection of *C. annuum* accessions from the principal areas of diversification. Parameters related to size and shape arose as the most informative and likely to be those suffering from the most intensive human selections after domestication. These digital attributes also served to corroborate the proposed differentiation of pepper fruits from the ancestor *C. annuum* var. *glabriusculum* to the most common bell type prevailing in current occidental markets. Fruit phenotypic data revealed that peppers from the Canary Islands and the most southeastern part of Spain possessed the highest similarities with American accessions, suggesting that ancient populations from America might have entered the South of Spain in post-Columbian times and promptly migrated to Portugal and the Mediterranean Basin. The current work can contribute to increasing the value of Iberian landraces, most of which persist in cultivation, favoring the breeding of additional cultivars with PDO. Lastly, the highly accurate fruit phenotyping can allow more reliable genome-wide association studies, which are often hampered by the shortage and poor quality of morphological descriptors.

## Figures and Tables

**Table 1:** Analysis of variance (sum of squares, mean squares, F-value, and R-square), mean, range, and coefficient of variation for conventional (FWE and FPL) and Tomato Analyzer descriptors in 139 *C. annuum* accessions. See Table S5 for trait acronyms.

Trait	Sum of Squares	Mean Squares	F-Value ^†^	R-Square	Mean	Range	%CV
FWE	536.75	4.87	82.62 *	0.93	59.12	0.10–404.30	117.3
FPL	536.64	4.87	23.70 *	0.80	3.99	0.90–9.40	31.5
P	454.07	4.14	80.18 *	0.93	233.10	28.02–662.51	47.7
A	670.19	6.15	56.37 *	0.90	2267.03	51.95–9451.79	72.4
WMH	454.19	4.14	70.69 *	0.92	38.05	3.89–164.59	63.3
MW	466.73	4.25	75.42 *	0.93	44.83	5.33–175.09	54.9
HMW	380.06	3.46	61.81 *	0.91	68.46	5.16–258.91	59.9
MH	393.84	3.58	87.42 *	0.94	77.30	9.90–260.18	58.8
CH	413.08	3.75	77.62 *	0.93	82.07	11.59–266.25	54.5
FSIEI	237.79	2.16	57.30 *	0.91	2.17	0.23–8.60	68.5
FSIEII	204.09	1.85	52.31 *	0.90	2.68	0.20–15.75	88.6
CFSI	184.57	1.69	40.96 *	0.87	3.17	0.27–20.86	86.3
PFB	249.49	2.27	8.22 *	0.55	0.96	0.17–2.29	30.4
DFB	156.92	1.42	3.71 *	0.32	0.59	0.08–2.00	31.7
FST	210.61	1.91	6.08 *	0.46	1.79	0.27–10.94	45.6
E	205.09	1.86	9.26 *	0.58	0.10	0.03–0.28	37.2
C	427.10	3.88	54.32 *	0.90	0.26	0.03–0.47	45.6
R	224.69	2.04	5.15 *	0.41	0.45	0.05–0.71	22.2
SH	148.57	1.35	2.27 *	0.17	0.03	0.00–0.30	133.2
PAMI	160.14	1.45	1.88 *	0.13	180.64	79.10–359.50	30.7
PAMA	389.75	3.54	7.15 *	0.51	151.81	37.90–351.00	41.8
DAMI	250.44	2.27	4.16 *	0.35	147.43	62.10–358.20	44.8
DAMA	321.64	2.92	7.27 *	0.51	93.38	10.80–358.00	72.0
Ov	270.72	2.46	7.74 *	0.53	0.33	0.00–0.87	59.2
VAs	138.18	1.26	6.64 *	0.49	0.36	0.01–2.54	92.2
HAov	253.51	2.31	24.39 *	0.80	0.83	0.00–6.28	106.2
WWP	210.56	1.91	6.30 *	0.47	0.32	0.03–0.97	54.2
EC	249.87	2.27	6.74 *	0.49	0.71	0.06–0.80	11.0
FSII	180.91	1.64	54.93 *	0.90	2.65	0.20–14.06	89.0
ECA	123.01	1.11	6.36 *	0.48	0.45	0.004–0.97	21.5
LD	222.93	2.03	60.51 *	0.91	25.78	1.47–129.78	87.5
PA	180.49	1.64	41.59 *	0.87	0.45	0.22–1.82	35.4
PT	157.06	1.43	5.18 *	0.41	0.21	0.07–0.54	14.9
AR	381.90	3.47	11.33 *	0.64	167.24	105.87–222.65	12.6
AG	446.34	4.05	12.76 *	0.67	86.52	38.47–201.75	25.4
AB	384.49	3.49	10.54 *	0.62	46.14	16.88–123.25	32.3
AL	391.03	3.55	9.24 *	0.58	100.40	64.04–159.36	13.3
ALV	424.19	3.86	12.03 *	0.65	46.40	27.88–81.03	15.9
AaV	449.33	4.08	16.19 *	0.72	26.44	0.33–41.55	27.7
AbV	422.19	3.83	19.30 *	0.76	39.65	18.94–68.48	18.9
AHue	478.73	4.35	21.05 *	0.77	56.68	44.44–101.97	14.8
ACh	362.11	3.30	16.38 *	0.73	48.03	23.87–69.14	17.8

^†^,* Significant at *p* < 0.0001.

## Data Availability

The data presented in this study are available on request from the corresponding author.

## Supplementary Materials

The following supporting information can be downloaded at https://www.mdpi.com/article/10.3390/plants11223075/s1, Table S1. Analysis of variance for conventional (FWE and FLP) and Tomato Analyzer descriptors among Mexico, Andean Region, Iberian Peninsula, and Mediterranean Basin; Table S2. Analysis of variance for conventional (FWE and FLP) and Tomato Analyzer descriptors among Iberian biogeographic provinces; Table S3. Analysis of variance for conventional and Tomato Analyzer descriptors between pungent and non-pungent peppers. Only the significantly different parameters are shown. FEW = fruit weight, FPL = fruit pedicel length; see Table S5 for acronyms of TA descriptors; Table S4. Scores of each pepper accession for the first (PC1) and second (PC2) components after PCA analysis. P = pungent, NP = non-pungent, *na* = not available; Table S5. List of accessions employed in this work. *na* stands for not available; Table S6. List of conventional descriptors (FWE and FPL) and digital traits measured using Tomato Analyzer v 3 software; Figure S1. Pearson’s rank correlation coefficients between pairs of traits. Color intensity (right scale) and the size of circles are proportional to the correlation coefficients. Significant and non-significant correlations are shown; Figure S2. Contribution coefficients of each trait to the two principal components (PCs); Figure S3. Biogeographic provinces of the Iberian Peninsula. Modified from Rivas-Martinez et al. [10].
