# Supplementary material for: Tracing Back the History of Pepper (Capsicum annuum) in the Iberian Peninsula from a Phenomics Point of View
Source: Plants (Basel). 2022 Nov 13;11(22):3075. doi: 10.3390/plants11223075 (PMC9699223; doi:10.3390/plants11223075)
Supplement: Supplementary file 1 [file plants-11-03075-s001.zip › plants-1865485-supplementary.pdf]

Table S1: Analysis of variance for conventional (FWE, FLP) and Tomato Analyzer descriptors among Mexico, Andean Region, Iberian Peninsula and Mediterranean Basin.

Trait	Acronym	Sum of squares	Mean squares	F value[†]
Fresh weight	FWE	134.02	44.67	66.13***
Fruit pedicel lenght	FPL	32.72	10.91	11.41***
Perimeter	P	101.96	33.99	57.56***
Area	A	140.84	46.95	51.13***
Width mid-height	WMH	152.56	50.86	98.22**
Maximum width	MW	156.21	52.07	98.28***
Height mid-width	HMW	46.80	15.60	27.62***
Maximum height	MH	53.94	17.98	31.95***
Curved height	CH	59.26	19.76	33.45***
Fruit shape index external I	FSIEI	37.89	12.63	36.90***
Fruit shape index external II	FSIEII	27.09	9.03	29.63***
Curved fruit shape index	CFSI	25.06	8.36	29.41***
Proximal fruit blockiness	PFB	12.97	4.33	7.23***
Distal fruit blockiness	DFB	21.93	7.31	13.79***
Fruit shape triangle	FST	27.07	9.02	16.46***
Ellipsoid	E	9.50	3.17	6.71***
Circular	C	98.03	32.68	57.19***
Rectangular	R	46.27	15.43	23.37***
Shoulder height	SH	14.84	4.95	7.03***
Proximal angle micro	PAMI	4.83	1.61	1.82^{ns}
Proximal angle macro	PAMA	89.37	29.79	33.85***
Distal angle micro	DAMI	18.00	6.00	7.34***
Distal angle macro	DAMA	46.94	15.65	20.52***
Ovoid	Ov	19.82	6.61	10.08***
V.Asymmetry	VAs	13.81	4.60	13.11***
H.Asymmetry.Ov	HAov	20.03	6.68	15.11***
Width widest position	WWP	12.77	4.26	7.59***
Eccentricity	EC	30.23	10.08	16.19***
Fruit shape index internal	FSII	25.17	8.39	31.41***
Eccentricity area index	ECA	5.26	1.75	5.32**
Lobedness degree	LD	32.62	10.87	33.57***
Pericarp area	PA	39.97	13.32	52.99***
Pericarp thickness	PT	14.09	4.70	10.40***
Average Red	AR	23.51	7.84	9.64***
Average Green	AG	6.54	2.18	2.29^{ns}
Average Blue	AB	26.11	8.70	10.44***
Average Luminosity	AL	10.67	3.56	3.90**
Average L Value	ALV	11.03	3.68	4.04**
Average a Value	AaV	8.77	2.93	3.26*
Average b Value	AbV	52.87	17.62	23.81***
Average Hue	AHue	4.81	1.60	1.76^{ns}
Average Chroma	ACh	45.81	15.27	23.15***

[†]***significant at $p < 0.001$, ** $p < 0.01$, * $p < 0.05$, ^{ns} not significant

Table S2: Analysis of variance for conventional (FWE, FLP) and Tomato Analyzer descriptors among Iberian biogeographic provinces.

Trait	Acronym	Sum of squares	Mean squares	F value†
Fresh weight	FWE	40.83	5.83	7.72***
Fruit pedicel lenght	FPL	33.04	4.72	4.57***
Perimeter	P	49.73	7.10	13.82***
Area	A	84.52	12.07	12.85***
Width mid-height	WMH	18.93	2.71	5.33***
Maximum width	MW	22.50	3.21	6.46***
Height mid-width	HMW	50.17	7.17	14.58***
Maximum height	MH	52.45	7.49	14.98***
Curved height	CH	53.00	7.57	14.27***
Fruit shape index external I	FSIEI	8.90	1.27	4.71***
Fruit shape index external II	FSIEII	4.68	0.67	3.08**
Curved fruit shape index	CFSI	4.24	0.61	3.17**
Proximal fruit blockiness	PFB	17.24	2.46	5.55***
Distal fruit blockiness	DFB	16.09	2.30	5.63***
Fruit shape triangle	FST	10.04	1.43	2.27*
Ellipsoid	E	17.20	2.46	5.27***
Circular	C	23.97	3.42	7.11***
Rectangular	R	2.13	0.30	0.56^{ns}
Shoulder height	SH	18.58	2.65	4.44***
Proximal angle micro	PAMI	12.29	1.76	2.10*
Proximal angle macro	PAMA	15.54	2.22	2.21*
Distal angle micro	DAMI	24.14	3.45	4.91***
Distal angle macro	DAMA	26.27	3.75	4.88***
Ovoid	Ov	23.02	3.29	6.20***
V.Asymmetry	VAs	10.72	1.53	5.82***
H.Asymmetry.Ov	HAov	20.47	2.93	8.29***
Width widest position	WWP	19.32	2.76	5.85***
Eccentricity	EC	27.36	3.91	4.04***
Fruit shape index internal	FSII	4.90	0.70	3.29**
Eccentricity area index	ECA	7.28	1.04	3.09**
Lobedness degree	LD	6.87	0.98	3.74**
Pericarp area	PA	17.89	2.56	9.78***
Pericarp thickness	PT	33.19	4.74	7.58***
Average Red	AR	74.35	10.62	18.04***
Average Green	AG	43.19	6.17	6.93***
Average Blue	AB	23.29	3.33	3.89***
Average Luminosity	AL	61.24	8.75	11.27***
Average L Value	ALV	66.16	9.45	12.70***
Average a Value	AaV	12.11	1.73	2.17*
Average b Value	AbV	61.69	8.81	14.93***
Average Hue	AHue	16.10	2.30	2.92**
Average Chroma	ACh	49.92	7.13	12.50***

†,***significant at $p < 0.001$, ** $p < 0.001$, * $p < 0.00$, ^{ns} not significant

Table S3: Analysis of variance for conventional and Tomato Analyzer descriptors between pungent and non-pungent peppers. Only the significantly different parameters are shown.

Trait	Acronym	Pungent		Non-pungent		Sig.
		Mean	CV(%)	Mean	CV(%)	
Fresh weight	FWE	18.58	123.19	63.27	91.85	<0.001
Fruit pedicel lenght	FPL	3.93	35.12	4.03	26.85	<0.001
Perimeter	P	205.10	60.51	254.21	34.81	<0.001
Area	A	1600.66	80.15	3029.77	56.58	<0.001
Width mid-height	WMH	21.24	63.17	41.09	45.85	<0.001
Maximum width	MW	27.95	52.44	48.08	37.24	<0.001
Height mid-width	HMW	72.70	67.47	79.66	45.08	<0.001
Maximum height	MH	81.74	67.46	90.08	43.17	<0.001
Curved height	CH	83.11	65.62	93.08	41.59	<0.001
Fruit shape index external I	FSIEI	3.07	54.17	2.12	50.25	<0.001
Fruit shape index external II	FSIEII	4.10	70.62	2.47	66.25	<0.001
Curved fruit shape index	CFSI	4.72	71.11	2.91	69.05	<0.001
Ellipsoid	E	0.10	44.18	0.10	34.11	0.006
Circular	C	0.31	42.83	0.25	45.40	0.001
Rectangular	R	0.41	25.70	0.46	20.47	<0.001
Shoulder height	SH	0.02	141.30	0.03	116.13	<0.001
Proximal angle micro	PAMI	135.35	49.09	160.80	42.49	<0.001
V.Asymmetry	VAs	0.27	97.30	0.31	75.96	<0.001
H.Asymmetry.Ov	HAov	1.06	97.32	1.00	78.51	<0.001
Eccentricity	EC	0.73	10.39	0.71	11.05	<0.001
Fruit shape index internal	FSII	4.02	72.24	2.47	66.15	<0.001
Eccentricity area index	ECA	0.48	14.76	0.48	13.84	0.003
Lobedness degree	LD	37.99	73.62	23.83	71.07	0.005
Pericarp area	PA	0.40	15.87	0.37	15.65	<0.001
Pericarp thickness	PT	0.21	15.81	0.20	16.11	<0.001
Average Red	AR	175.64	10.47	167.61	10.72	<0.001
Average Green	AG	90.37	29.50	83.41	20.84	<0.001
Average Luminosity	AL	103.50	13.27	99.02	12.29	0.003
Average L Value	ALV	48.59	16.85	45.72	13.12	<0.001
Average b Value	AbV	43.15	13.41	40.60	11.96	<0.001
Average Hue	AHue	57.75	17.33	55.90	10.92	<0.001
Average Chroma	ACh	51.87	10.80	49.42	11.74	<0.001

Table S4 : Scores of each pepper accession for the first (PC1) and second (PC2) components after PCA analysis. P = pungent, NP = non-pungent, *na* = not available.

Accession	Country	Region	Pungency	PC1	PC2
C204	Mexico	Mexico	P	-0.9439	-1.6398
C87	Mexico	Mexico	NP	0.4309	-0.8216
CGN21460	Mexico	Mexico	P	0.5216	-1.8411
CGN21526	Mexico	Mexico	NP	0.1372	-1.2737
CGN21527	Mexico	Mexico	P	0.7564	-1.4166
CGN22102	Mexico	Mexico	P	0.2853	-0.7072
CGN22783	Mexico	Mexico	P	-0.1551	-1.8769
CGN23254	Mexico	Mexico	P	-0.3293	0.1655
CGN24355	Mexico	Mexico	NP	1.6527	-0.2887
PM647	Mexico	Mexico	P	-0.0180	-2.1073
BGV005857	Ecuador	Andean Region	NP	0.5611	0.0951
BGV005981	Ecuador	Andean Region	NP	0.1819	0.0278
BGV006008	Ecuador	Andean Region	NP	-0.1033	0.0979
BGV006055	Ecuador	Andean Region	NP	0.6901	-0.0095
PI241670	Ecuador	Andean Region	NP	2.4641	-1.2090
PI585238	Ecuador	Andean Region	P	3.1917	-1.5304
PI585246	Ecuador	Andean Region	P	0.4181	-0.2001
CGN20808	Bolivia	Andean Region	P	0.0226	-2.1433
CGN22155	Bolivia	Andean Region	NP	0.1256	-0.8361
PI213915	Bolivia	Andean Region	NP	1.3633	-1.0182
PI439212	Bolivia	Andean Region	P	1.6737	-0.9516
PI543188	Bolivia	Andean Region	P	-0.0477	-0.3436
BGV013687	Peru	Andean Region	NP	-0.2091	-0.0468
BGV013691	Peru	Andean Region	NP	0.2316	-0.3378
CAP524	Peru	Andean Region	P	-0.3387	-1.8026
BGHZ0181	Spain	Iberian Peninsula	P	-0.5887	-0.2256
BGHZ0284	Spain	Iberian Peninsula	P	2.5758	-0.2153
BGHZ1109	Spain	Iberian Peninsula	NP	-0.8327	0.9390
BGHZ2154	Spain	Iberian Peninsula	P	1.7161	0.2499
BGHZ2546	Spain	Iberian Peninsula	NP	-1.1624	0.7405
BR11305	Spain	Iberian Peninsula	NP	0.0667	-0.0430
OI2705	Spain	Iberian Peninsula	NP	0.0527	1.9436
PA145	Spain	Iberian Peninsula	NP	-0.5721	0.1380
PI30905	Spain	Iberian Peninsula	NP	-0.7576	0.9654
BPGV13044	Portugal	Iberian Peninsula	*na*	0.0478	-0.6770
BPGV13059	Portugal	Iberian Peninsula	*na*	-0.0231	-1.1700
BPGV16049	Portugal	Iberian Peninsula	*na*	0.1108	0.1901
BPGV16134	Portugal	Iberian Peninsula	*na*	-0.1619	-0.7993
BPGV16135	Portugal	Iberian Peninsula	*na*	0.4197	1.5745
BPGV16137	Portugal	Iberian Peninsula	*na*	0.0642	-1.5937
BPGV16184	Portugal	Iberian Peninsula	*na*	-0.3617	-0.1784
BGHZ0106	Spain	Iberian Peninsula	P	1.5523	0.5226
BGHZ0113	Spain	Iberian Peninsula	*na*	-0.2514	0.9498
BGHZ0179	Spain	Iberian Peninsula	P	-0.7215	0.0786
BGHZ1375	Spain	Iberian Peninsula	P	1.1655	0.2085
BGHZ1521	Spain	Iberian Peninsula	P	1.4961	1.6038
BGHZ1753	Spain	Iberian Peninsula	P	1.6643	-0.0481
BGHZ2561	Spain	Iberian Peninsula	P	-1.4771	0.0678
BPGV07150	Portugal	Iberian Peninsula	*na*	-0.0250	-0.0817
BPGV12297	Portugal	Iberian Peninsula	*na*	-0.0941	-0.6735
BPGV12303	Portugal	Iberian Peninsula	*na*	-0.2474	0.6874
BPGV12306	Portugal	Iberian Peninsula	*na*	-0.4286	-0.4322
BPGV12330	Portugal	Iberian Peninsula	*na*	-0.3426	2.2467
BPGV12331	Portugal	Iberian Peninsula	*na*	-0.3696	0.0490
BPGV12350	Portugal	Iberian Peninsula	*na*	0.0738	-0.1164
BPGV12351	Portugal	Iberian Peninsula	*na*	-0.0021	-0.8439
BPGV12353	Portugal	Iberian Peninsula	*na*	-0.0770	1.3930
BPGV12354	Portugal	Iberian Peninsula	*na*	0.2288	0.4379
BPGV12381	Portugal	Iberian Peninsula	*na*	-0.0939	1.3355
BPGV12387	Portugal	Iberian Peninsula	*na*	-0.4616	0.2397
BPGV12417	Portugal	Iberian Peninsula	*na*	0.0943	-0.2953
BPGV12438	Portugal	Iberian Peninsula	*na*	-0.1409	0.8240
BPGV12440	Portugal	Iberian Peninsula	*na*	-0.4259	-0.5871
BPGV16393	Portugal	Iberian Peninsula	*na*	-0.0504	-0.5772
CGN21475	Portugal	Iberian Peninsula	NP	-1.2594	-1.3967
BGHZ0206	Spain	Iberian Peninsula	NP	-0.7532	1.8714
CGN23293	Spain	Iberian Peninsula	NP	-1.1687	1.5185
BPGV12903	Portugal	Iberian Peninsula	*na*	0.1158	1.5608
BPGV12904	Portugal	Iberian Peninsula	*na*	-0.2719	-0.0950

CAP1371	Portugal	Iberian Peninsula	NP	-0.4714	0.1660
CAP1539	Portugal	Iberian Peninsula	NP	0.0313	-1.8218
CGN16805	Portugal	Iberian Peninsula	NP	-0.8868	-0.0093
CGN16897	Portugal	Iberian Peninsula	NP	-1.5136	0.9166
CGN16899	Portugal	Iberian Peninsula	NP	-1.1395	-1.2251
CGN22161	Portugal	Iberian Peninsula	NP	-0.8524	0.6233
BGHZ0097	Spain	Iberian Peninsula	P	0.3598	0.2322
BGHZ0102	Spain	Iberian Peninsula	NP	-0.6495	1.0257
BGHZ0224	Spain	Iberian Peninsula	NP	-1.3381	0.0035
BGHZ0280	Spain	Iberian Peninsula	P	-1.1532	-1.8042
BGHZ0447	Spain	Iberian Peninsula	NP	1.2405	-0.1912
BGHZ0775	Spain	Iberian Peninsula	NP	-0.6583	1.3669
BGHZ0782	Spain	Iberian Peninsula	P	-0.5356	-1.1939
BGHZ0969	Spain	Iberian Peninsula	P	-1.0440	-1.5168
BGHZ1174	Spain	Iberian Peninsula	NP	-1.2481	0.1520
BGHZ0213	Spain	Iberian Peninsula	NP	0.1033	1.6332
BGHZ0337	Spain	Iberian Peninsula	NP	-1.1510	0.6828
BGHZ3337	Spain	Iberian Peninsula	NP	1.4544	1.4996
CAP1661	Spain	Iberian Peninsula	NP	-0.1475	1.6375
CGN17234	Spain	Iberian Peninsula	NP	1.0504	1.6964
CGN17235	Spain	Iberian Peninsula	P	1.0672	-1.0490
BGHZ0413	Spain	Iberian Peninsula	NP	-0.2696	0.9513
BGHZ0463	Spain	Iberian Peninsula	NP	-0.8212	-0.8484
BGHZ5912	Spain	Iberian Peninsula	NP	-1.2209	0.6621
C431	Spain	Iberian Peninsula	NP	-1.2681	-0.8138
BGHZ0282	Spain	Iberian Peninsula	NP	1.0640	1.2229
BGHZ0338	Spain	Iberian Peninsula	NP	-0.3093	1.1783
BGHZ0971	Spain	Iberian Peninsula	NP	-0.3618	0.8301
CGN20507	Spain	Iberian Peninsula	NP	-0.3006	1.7148
BGHZ0082	Spain	Iberian Peninsula	P	-1.0243	-1.2294
BGHZ1114	Spain	Iberian Peninsula	P	0.3481	-1.6557
CAP1049	Albania	Mediterranean Basin	NP	0.8548	1.0897
CAP1051	Albania	Mediterranean Basin	P	1.6981	0.1008
CGN17172	France	Mediterranean Basin	NP	0.8087	1.8655
CGN21486	France	Mediterranean Basin	NP	-1.1969	1.4907
CGN23239	France	Mediterranean Basin	NP	0.9903	-0.1447
CAP242	Italy	Mediterranean Basin	NP	-0.4315	0.0018
CAP68	Italy	Mediterranean Basin	NP	-1.3251	-0.6018
CAP913	Italy	Mediterranean Basin	NP	-1.0438	1.1821
CGN16800	Italy	Mediterranean Basin	P	-1.2873	-1.3802
CGN16881	Italy	Mediterranean Basin	P	-1.2617	0.3213
CGN16916	Israel	Mediterranean Basin	NP	-0.7177	-0.2873
CGN16917	Israel	Mediterranean Basin	NP	-0.6662	0.2914
CGN21480	Israel	Mediterranean Basin	NP	-0.7064	-0.1538
CGN22825	Israel	Mediterranean Basin	P	1.5128	0.0076
CAP607	Libya	Mediterranean Basin	*na*	-0.1559	-0.3009
CAP609	Libya	Mediterranean Basin	P	0.8743	0.4063
CAP614	Libya	Mediterranean Basin	P	0.2639	-1.1400
CAP1538	Tunisia	Mediterranean Basin	P	-0.6362	0.1754
CAP977	Tunisia	Mediterranean Basin	P	-0.3081	-0.8422
CAP979	Tunisia	Mediterranean Basin	P	0.4457	0.8833
CGN16833	Turkey	Mediterranean Basin	NP	-0.8674	-0.0631
CGN17010	Turkey	Mediterranean Basin	NP	-0.8351	-0.0521
CGN17190	Turkey	Mediterranean Basin	NP	-0.6797	0.2694
CGN17191	Turkey	Mediterranean Basin	NP	1.5187	0.8229
CGN22154	Turkey	Mediterranean Basin	P	-0.9660	0.0406
CGN24358	Turkey	Mediterranean Basin	P	2.1735	0.5872
CAP1254	Bulgaria	Mediterranean Basin	P	0.9354	-1.1778
CAP491	Bulgaria	Mediterranean Basin	P	2.0776	0.4220
CGN16808	Bulgaria	Mediterranean Basin	*na*	0.0500	0.2571
CGN17214	Bulgaria	Mediterranean Basin	NP	-1.1886	-0.6324
CGN21581	Slovenia	Mediterranean Basin	NP	0.4703	-0.0558
CGN16807	Hungary	Mediterranean Basin	NP	-1.3876	-0.6403
CGN16817	Hungary	Mediterranean Basin	*na*	-1.0264	-0.5113
CGN17147	Hungary	Mediterranean Basin	NP	-1.2115	-0.6580
CGN17227	Hungary	Mediterranean Basin	*na*	-0.5466	0.1018
CGN22127	Hungary	Mediterranean Basin	P	1.4388	0.2550
CGN16835	Former Yugoslavia	Mediterranean Basin	P	1.9945	1.0214
CGN17193	Former Yugoslavia	Mediterranean Basin	NP	1.1341	1.0858
CGN23235	Former Yugoslavia	Mediterranean Basin	NP	-1.1451	1.1546

Table S5 : List of accessions employed in this work. *Na* stands for not available.

Accession	Country	Collection site	Geographical Region	Biogeographic Province[a]	Population type [b]	Pungency [c]	Source [d]
BGHZ0082	Spain	Tenerife	Iberian Peninsula	CI	land variety	P	BGHZ
BGHZ0097	Spain	Madrid	Iberian Peninsula	CIM	land variety	P	BGHZ
BGHZ0102	Spain	Lerida	Iberian Peninsula	CIM	land variety	NP	BGHZ
BGHZ0106	Spain	Caceres	Iberian Peninsula	WIM	land variety	P	BGHZ
BGHZ0113	Spain	Badajoz	Iberian Peninsula	WIM	land variety	*na*	BGHZ
BGHZ0179	Spain	Zamora	Iberian Peninsula	WIM	land variety	P	BGHZ
BGHZ0181	Spain	Vizcaya	Iberian Peninsula	EA	land variety	P	BGHZ
BGHZ0206	Spain	Huelva	Iberian Peninsula	CLWA	land variety	NP	BGHZ
BGHZ0213	Spain	Mallorca	Iberian Peninsula	VPB	land variety	NP	BGHZ
BGHZ0224	Spain	La Rioja	Iberian Peninsula	CIM	land variety	NP	BGHZ
BGHZ0280	Spain	Toledo	Iberian Peninsula	CIM	land variety	P	BGHZ
BGHZ0282	Spain	Jaen	Iberian Peninsula	B	land variety	NP	BGHZ
BGHZ0284	Spain	Guipuzcoa	Iberian Peninsula	EA	land variety	P	BGHZ
BGHZ0337	Spain	Valencia	Iberian Peninsula	VPB	land variety	NP	BGHZ
BGHZ0338	Spain	Malaga	Iberian Peninsula	B	land variety	NP	BGHZ
BGHZ0413	Spain	Almeria	Iberian Peninsula	MA	land variety	NP	BGHZ
BGHZ0447	Spain	Teruel	Iberian Peninsula	CIM	land variety	NP	BGHZ
BGHZ0463	Spain	Murcia	Iberian Peninsula	MA	land variety	NP	BGHZ
BGHZ0775	Spain	Albacete	Iberian Peninsula	CIM	land variety	NP	BGHZ
BGHZ0782	Spain	Ciudad Real	Iberian Peninsula	CIM	land variety	P	BGHZ
BGHZ0969	Spain	Guadalajara	Iberian Peninsula	CIM	land variety	P	BGHZ
BGHZ0971	Spain	Granada	Iberian Peninsula	B	land variety	NP	BGHZ
BGHZ1109	Spain	Asturias	Iberian Peninsula	EA	land variety	NP	BGHZ
BGHZ1114	Spain	Las Palmas	Iberian Peninsula	CI	land variety	P	BGHZ
BGHZ1174	Spain	Zaragoza	Iberian Peninsula	CIM	land variety	NP	BGHZ
BGHZ1375	Spain	Avila	Iberian Peninsula	WIM	land variety	P	BGHZ
BGHZ1521	Spain	Caceres	Iberian Peninsula	WIM	land variety	P	BGHZ
BGHZ1753	Spain	Segovia	Iberian Peninsula	WIM	land variety	P	BGHZ
BGHZ2154	Spain	Cantabria	Iberian Peninsula	EA	land variety	P	BGHZ
BGHZ2546	Spain	Navarra	Iberian Peninsula	EA	land variety	NP	BGHZ
BGHZ2561	Spain	Leon	Iberian Peninsula	WIM	land variety	P	BGHZ
BGHZ3337	Spain	Ibiza	Iberian Peninsula	VPB	land variety	NP	BGHZ
BGHZ5912	Spain	Alicante	Iberian Peninsula	MA	land variety	NP	BGHZ
BR11305	Spain	Pontevedra	Iberian Peninsula	EA	land variety	NP	CIAM
C431	Spain	Murcia	Iberian Peninsula	MA	land variety	NP	BGHZ
CAP1661	Spain	Barcelona	Iberian Peninsula	VPB	land variety	NP	IPK
CGN17234	Spain	Valencia	Iberian Peninsula	VPB	land variety	NP	CGN
CGN17235	Spain	Castellon	Iberian Peninsula	VPB	land variety	P	CGN
CGN20507	Spain	Córdoba	Iberian Peninsula	B	land variety	NP	CGN
CGN23293	Spain	Cadiz	Iberian Peninsula	CLWA	land variety	NP	CGN
OI2705	Spain	Orense	Iberian Peninsula	EA	land variety	NP	CIAM
PA145	Spain	A Coruña	Iberian Peninsula	EA	land variety	NP	CIAM
PI30905	Spain	Lugo	Iberian Peninsula	EA	land variety	NP	CIAM
BPGV07150	Portugal	Evora	Iberian Peninsula	WIM	land variety	*na*	BPGV
BPGV12297	Portugal	Braganca	Iberian Peninsula	WIM	land variety	*na*	BPGV
BPGV12303	Portugal	Braganca	Iberian Peninsula	WIM	land variety	*na*	BPGV
BPGV12306	Portugal	Braganca	Iberian Peninsula	WIM	land variety	*na*	BPGV
BPGV12330	Portugal	Braganca	Iberian Peninsula	WIM	land variety	*na*	BPGV
BPGV12331	Portugal	Braganca	Iberian Peninsula	WIM	land variety	*na*	BPGV
BPGV12350	Portugal	Braganca	Iberian Peninsula	WIM	land variety	*na*	BPGV
BPGV12351	Portugal	Braganca	Iberian Peninsula	WIM	land variety	*na*	BPGV
BPGV12353	Portugal	Braganca	Iberian Peninsula	WIM	land variety	*na*	BPGV
BPGV12354	Portugal	Braganca	Iberian Peninsula	WIM	land variety	*na*	BPGV
BPGV12381	Portugal	Braganca	Iberian Peninsula	WIM	land variety	*na*	BPGV
BPGV12387	Portugal	Braganca	Iberian Peninsula	WIM	land variety	*na*	BPGV
BPGV12417	Portugal	Braganca	Iberian Peninsula	WIM	land variety	*na*	BPGV
BPGV12438	Portugal	Braganca	Iberian Peninsula	WIM	land variety	*na*	BPGV
BPGV12440	Portugal	Braganca	Iberian Peninsula	WIM	land variety	*na*	BPGV
BPGV12903	Portugal	Aveiro	Iberian Peninsula	CLWA	land variety	*na*	BPGV
BPGV12904	Portugal	Aveiro	Iberian Peninsula	CLWA	land variety	*na*	BPGV
BPGV13044	Portugal	Guarda	Iberian Peninsula	EA	land variety	*na*	BPGV
BPGV13059	Portugal	Guarda	Iberian Peninsula	EA	land variety	*na*	BPGV
BPGV16049	Portugal	Guarda	Iberian Peninsula	EA	land variety	*na*	BPGV
BPGV16134	Portugal	Guarda	Iberian Peninsula	EA	land variety	*na*	BPGV
BPGV16135	Portugal	Guarda	Iberian Peninsula	EA	land variety	*na*	BPGV
BPGV16137	Portugal	Guarda	Iberian Peninsula	EA	land variety	*na*	BPGV
BPGV16184	Portugal	Guarda	Iberian Peninsula	EA	land variety	*na*	BPGV
BPGV16393	Portugal	Castelo Branco	Iberian Peninsula	WIM	land variety	*na*	BPGV
CAP1371	Portugal	Lisboa	Iberian Peninsula	CLWA	improved cultivar	NP	IPK
CAP1539	Portugal	Algarve	Iberian Peninsula	CLWA	land variety	NP	IPK
CGN16805	Portugal	Oeiras	Iberian Peninsula	CLWA	breeders variety	NP	CGN
CGN16897	Portugal	Oeiras	Iberian Peninsula	CLWA	breeders variety	NP	CGN

CGN16899	Portugal	Oeiras	Iberian Peninsula	CLWA	breeders variety	NP	CGN
CGN21475	Portugal	Elvas	Iberian Peninsula	WIM	breeders variety	NP	CGN
CGN22161	Portugal	Oeiras	Iberian Peninsula	CLWA	breeders variety	NP	CGN
CAP1049	Albania	Shkallnur	Mediterranean Basin	-	land variety	NP	IPK
CAP1051	Albania	Shkallnur	Mediterranean Basin	-	land variety	P	IPK
CAP1254	Bulgaria	*na*	Mediterranean Basin	-	improved cultivar	P	IPK
CAP491	Bulgaria	*na*	Mediterranean Basin	-	improved cultivar	P	IPK
CGN16808	Bulgaria	*na*	Mediterranean Basin	-	*na*	*na*	CGN
CGN17214	Bulgaria	*na*	Mediterranean Basin	-	*na*	NP	CGN
CGN21581	Slovenia	*na*	Mediterranean Basin	-	breeders variety	NP	CGN
CGN16835	Former Yugoslavia	Ilovica	Mediterranean Basin	-	land variety	P	CGN
CGN17193	Former Yugoslavia	*na*	Mediterranean Basin	-	breeders variety	NP	CGN
CGN23235	Former Yugoslavia	*na*	Mediterranean Basin	-	*na*	NP	CGN
CGN17172	France	*na*	Mediterranean Basin	-	breeders variety	NP	CGN
CGN21486	France	*na*	Mediterranean Basin	-	breeders variety	NP	CGN
CGN23239	France	*na*	Mediterranean Basin	-	breeders variety	NP	CGN
CGN16807	Hungary	*na*	Mediterranean Basin	-	breeders variety	NP	CGN
CGN16817	Hungary	*na*	Mediterranean Basin	-	breeders variety	*na*	CGN
CGN17147	Hungary	*na*	Mediterranean Basin	-	breeders variety	NP	CGN
CGN17227	Hungary	*na*	Mediterranean Basin	-	land variety	*na*	CGN
CGN22127	Hungary	*na*	Mediterranean Basin	-	breeders variety	P	CGN
CGN16916	Israel	*na*	Mediterranean Basin	-	breeders variety	NP	CGN
CGN16917	Israel	*na*	Mediterranean Basin	-	breeders variety	NP	CGN
CGN21480	Israel	*na*	Mediterranean Basin	-	breeders variety	NP	CGN
CGN22825	Israel	*na*	Mediterranean Basin	-	breeders variety	P	CGN
CAP242	Italy	Aprigliano	Mediterranean Basin	-	land variety	NP	IPK
CAP68	Italy	Palinuro	Mediterranean Basin	-	land variety	NP	IPK
CAP913	Italy	Castellina Marittima	Mediterranean Basin	-	land variety	NP	IPK
CGN16800	Italy	*na*	Mediterranean Basin	-	breeders variety	P	CGN
CGN16881	Italy	*na*	Mediterranean Basin	-	breeders variety	P	CGN
CAP607	Libya	Az Zighan	Mediterranean Basin	-	land variety	*na*	IPK
CAP609	Libya	Tmassah	Mediterranean Basin	-	land variety	P	IPK
CAP614	Libya	Al Barkat	Mediterranean Basin	-	land variety	P	IPK
CAP1538	Tunisia	Fondouk-El-Aouareb	Mediterranean Basin	-	land variety	P	IPK
CAP977	Tunisia	Degache	Mediterranean Basin	-	land variety	P	IPK
CAP979	Tunisia	Qibilī	Mediterranean Basin	-	land variety	P	IPK
CGN16833	Turkey	*na*	Mediterranean Basin	-	land variety	NP	CGN
CGN17010	Turkey	*na*	Mediterranean Basin	-	breeders variety	NP	CGN
CGN17190	Turkey	*na*	Mediterranean Basin	-	breeders variety	NP	CGN
CGN17191	Turkey	*na*	Mediterranean Basin	-	breeders variety	NP	CGN
CGN22154	Turkey	Mugla	Mediterranean Basin	-	Land variety	P	CGN
CGN24358	Turkey	*na*	Mediterranean Basin	-	*na*	P	CGN
C204	Mexico	*na*	Mexico	-	land variety	P	BGHZ
C87	Mexico	Cihuatlán	Mexico	-	land variety	NP	BGHZ
CGN21460	Mexico	Tabasco	Mexico	-	land variety	P	CGN
CGN21526	Mexico	Ciudad de México	Mexico	-	*wild*	NP	CGN
CGN21527	Mexico	Puebla	Mexico	-	*wild*	P	CGN
CGN22102	Mexico	*na*	Mexico	-	land variety	P	CGN
CGN22783	Mexico	Tabasco	Mexico	-	*wild*	P	CGN
CGN23254	Mexico	*na*	Mexico	-	land variety	P	CGN
CGN24355	Mexico	Morelos	Mexico	-	land variety	NP	CGN
PM647	Mexico	*na*	Mexico	-	*wild*	P	INRAE
CGN20808	Bolivia	*na*	Andean Region	-	*wild*	P	CGN
CGN22155	Bolivia	Cochabamba	Andean Region	-	*na*	NP	CGN
PI213915	Bolivia	Cochabamba	Andean Region	-	*na*	NP	USDA
PI439212	Bolivia	*na*	Andean Region	-	*na*	P	USDA
PI543188	Bolivia	Cobija	Andean Region	-	cultivated material	P	USDA
BGV005857	Ecuador	Loja	Andean Region	-	*na*	NP	COMAV
BGV005981	Ecuador	Azuay	Andean Region	-	*na*	NP	COMAV
BGV006008	Ecuador	Chimborazo	Andean Region	-	*na*	NP	COMAV
BGV006055	Ecuador	Quito	Andean Region	-	*na*	NP	COMAV
PI241670	Ecuador	Guayaquil	Andean Region	-	*na*	NP	USDA
PI585238	Ecuador	Gonzanama	Andean Region	-	*na*	P	USDA
PI585246	Ecuador	Arenillas	Andean Region	-	*na*	P	USDA
BGV013687	Peru	Piura	Andean Region	-	*na*	NP	COMAV
BGV013691	Peru	Salta	Andean Region	-	*na*	NP	COMAV
CAP524	Peru	Lima	Andean Region	-	land variety	P	IPK

[a]EA = European Atlantic, BP = Betica Province, CIM = Central Iberian Mediterranean, CLWA = Coastal Lusitania and West Andalusia, MA = Murcia and Almeria, VPB = Valencia-Provence and Balearic, WIM = West Iberian Mediterranean, CI = Canary Islands

[b]Population type was assigned according to the information available at each genebank. *Na* stands for not available

[c]Pungency data were obtained from genebanks and previous work [59]. P = pungent, NP = non-pungent, *Na* stands for not available

[d]BGHZ=Vegetable Germplasm Bank of Zaragoza
BPGV=Portuguese Vegetal Germplasm Bank
COMAV= Instituto de Conservación y Mejora de la Agrodiversidad Valenciana,
CGN= Center for Genetic Resources,
IPK=Leibniz Institute of Plant Genetics and Crop Plant Research,
USDA= USDA - ARS - Plant Genetic Resources Conservation Unit
CIAM=Agricultural Research Center of Mabegondo
INRAE=French National Institute for Agriculture, Food, and Environment

Table S6: Conventional descriptors and digital traits measured with Tomato Analyzer v 3 software.

**Conventional descriptors**	**Acronym**	**Units**
Fruit weight	FWE	g
Fruit pedicel lenght	FPL	cm
**Tomato Analyzer Descriptors**		
**Basic Measurements:**		
Perimeter	P	mm
Area	A	mm^2
Width mid-height	WMH	mm
Maximum width	MW	mm
Height mid-width	HMW	mm
Maximum height	MH	mm
Curved height	CH	mm
**Fruit Shape Index:**		
Fruit shape index external I	FSIEI	
Fruit shape index external II	FSIEII	
Curved fruit shape index	CFSI	
**Blockiness:**		
Proximal fruit blockiness	PFB	
Distal fruit blockiness	DFB	
Fruit shape triangle	FST	
**Homogeneity:**		
Ellipsoid	E	
Circular	C	
Rectangular	R	
**Proximal Fruit End Shape:**		
Shoulder height	SH	
Proximal angle micro	PAMI	degrees
Proximal angle macro	PAMA	degrees
Proximal indentation area	PIA	
**Distal Fruit End Shape:**		
Distal angle micro	DAMI	degrees
Distal angle macro	DAMA	degrees
Distal indentation area	DIA	
Distal end protrusion	DEP	
**Asymmetry:**		
Obovoid	Ob	
Ovoid	Ov	
V.Asymmetry	VAs	
H.Asymmetry.Ob	HAob	
H.Asymmetry.Ov	HAov	
Width widest position	WWP	
**Internal Eccentricity:**		
Eccentricity	EC	
Proximal eccentricity	PEC	
Distal eccentricity	DEC	
Fruit shape index internal	FSII	
Eccentricity area index	ECA	
**Lattitudinal Section:**		
Lobedness degree	LD	
Pericarp area	PA	
Pericarp thickness	PT	

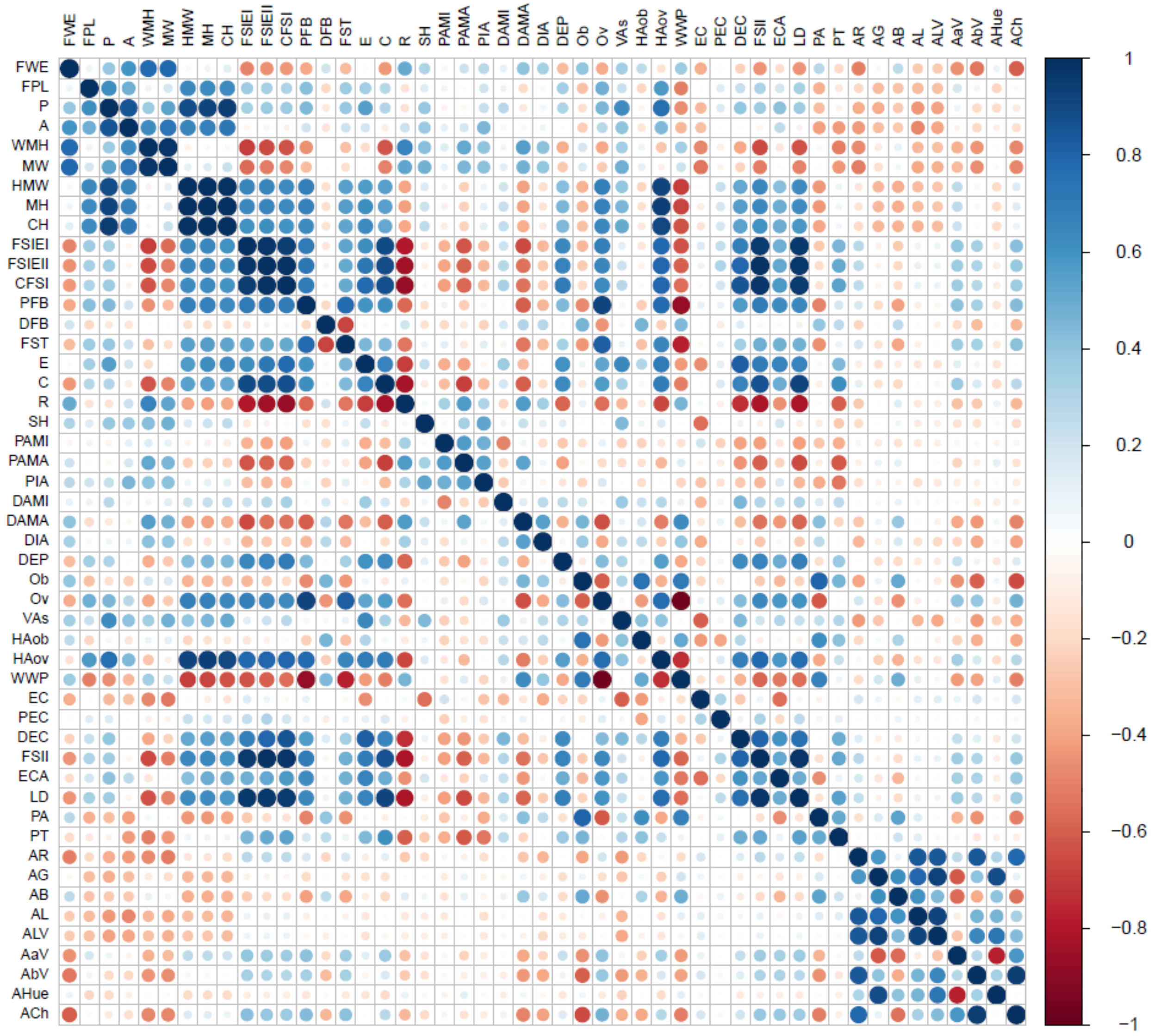

Figure S1: Pearson's rank correlation coefficients between pairs of traits. Color intensity (right scale) and the size of circles are proportional to the correlation coefficients. Significant and non-significant correlations are shown.

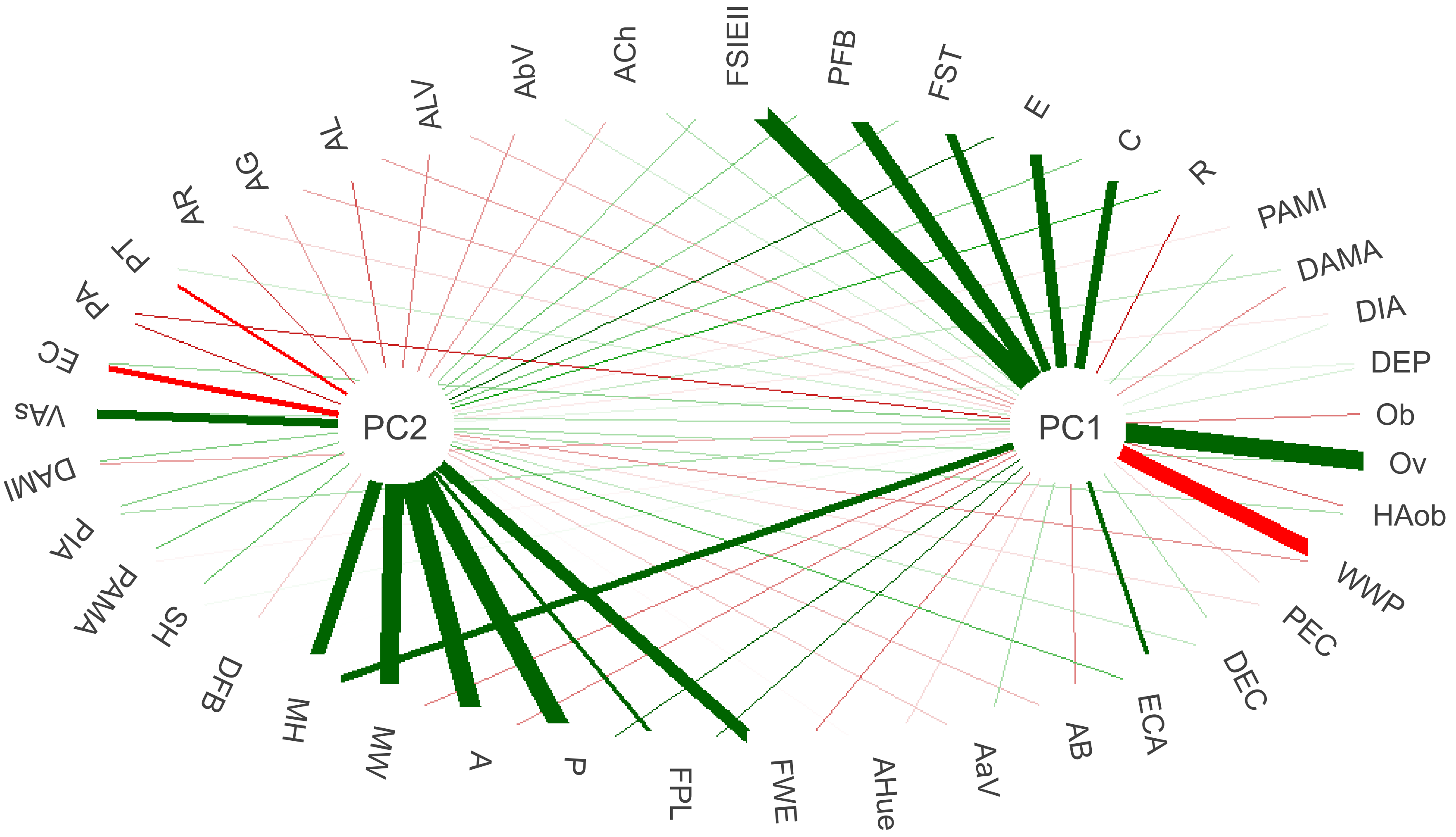

Figure S2: Contribution coefficients of each trait to the two Principal Components (PCs).

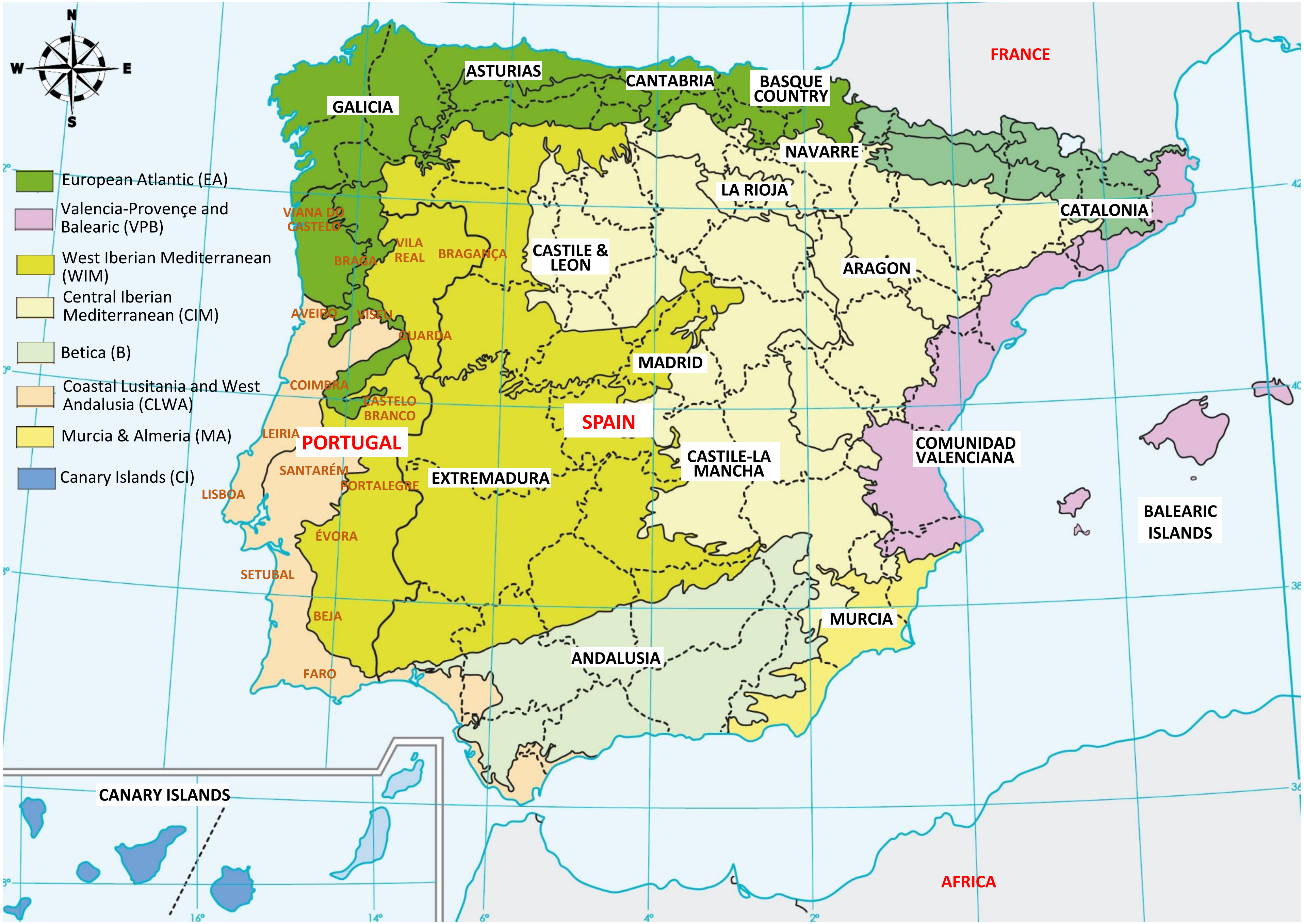

Figure S3: Biogeographic provinces of the Iberian Peninsula. Modified from Rivas-Martinez et al. [10]
